# Qualitative study exploring parenting among mothers and female caregivers living with the IPV, mental health and HIV syndemic in South Africa

**DOI:** 10.1136/bmjopen-2024-086478

**Published:** 2024-10-29

**Authors:** Mpho Silima, Nicola Joan Christofides, Hannabeth Franchino-Olsen, Nataly Woollett, Franziska Meinck

**Affiliations:** 1 School of Public Health, University of the Witwatersrand Johannesburg, Johannesburg, South Africa; 2 The Ohio State University, Columbus, Ohio, USA; 3 The University of Edinburgh School of Social and Political Science, Edinburgh, UK

**Keywords:** Caregivers, HIV & AIDS, MENTAL HEALTH

## Abstract

**Abstract:**

**Background:**

In South Africa, women disproportionately bear the burden of intimate partner violence (IPV), HIV or AIDS, and poor mental health.

**Objective:**

This study investigated parenting practices among women affected by IPV, HIV and poor mental health syndemics.

**Study setting:**

The study was conducted in two sites, a peri-urban area and a rural area in Mpumalanga, South Africa.

**Study design:**

A qualitative research design using a narrative approach with in-depth interviews supported by arts-based methods was used. Data were analysed thematically using MAXQDA (2022).

**Participants:**

20 women aged 20–60 who screened positive for HIV, IPV and/or poor mental health in a larger three-generational cohort study were selected.

**Results:**

Living with the syndemics exacerbated socioeconomic challenges that often translated into an inability to meet basic child needs. Socioeconomic challenges also led to more harsh parenting practices among women living with IPV-Mental Health and HIV-Mental Health syndemics. Due to lack of trust from family members, women living with the HIV-Mental Health-IPV syndemic were often separated from their children. These women exhibited less harsh parenting practices than the women in the other syndemic groups when they did see their children. A history of childhood trauma, leading to overprotective parenting, was common across the groups except for the IPV-Mental Health group. Women in the IPV-Mental Health group often had strained relations with their children’s fathers, affecting their engagement and connection with their children.

**Conclusion:**

The study underlines challenges experienced by women with IPV-Mental Health, HIV-Mental Health and HIV-Mental Health-IPV syndemics. The overlap of these epidemics strains women’s relationships and affects women’s parenting practices detrimentally resulting in an inadequate provision for children’s needs.

**Strengths and limitations of this study**

- The use of arts-based methods allowed for deeper and more detailed discussions and open conversations around highly sensitive topics.
- The quantitative study’s earlier initiation allowed trust to be established with participants a year before the qualitative study, making it easier to build rapport during the qualitative phase.
- Sensitive nature of the topics being discussed may have led to a lack of disclosure in some instances.
- Although validated scales for mental health were used to identify participants with poor mental health, this approach may have included individuals who endorsed symptoms but did not meet the strict cut-off criteria for a specific mental health challenge. This could have led to some misclassification.

## Introduction

 In South Africa, women disproportionately bear the burden of HIV, intimate partner violence (IPV) and poor mental health, which can significantly affect their parenting.^1^,^3^ Syndemic theory offers a lens to understand how these overlapping epidemics interact and amplify health burdens.^4^ This theory posits that multiple comorbidities, fuelled by structural and environmental factors, have a synergistic effect that worsens the overall health impact on a population more than the sum of individual conditions.^4^ Singer’s early work on syndemic theory primarily focused on the co-occurrence of substance abuse, violence and AIDS (SAVA) among marginalised communities in the USA.^4^ The current study expands on SAVA to include poor mental health. This expansion is not new, as Tsuyuki and colleagues studied the syndemic of substance use disorder, violence and mental health among HIV-infected individuals.^5^ However, very little research has investigated the impact of these syndemics on parenting practices.

### Conceptual framework: IPV, mental health and HIV as a syndemic

Existing literature confirms the frequent co-occurrence of IPV, poor mental health and HIV epidemics.^6^,^9^ The relationship between HIV and poor mental health is bidirectional, meaning that HIV can lead to poor mental health outcomes, and poor mental health can increase the risk of acquiring HIV.^10 11^ The high prevalence of poor mental health conditions among people living with HIV is often associated with poorer physical health, lower quality of health services and poor adherence to antiretroviral treatment.^12^,^14^ Furthermore, having poor mental health can increase the risk of HIV acquisition through both direct and indirect mechanisms.^15^ Adolescents and adults with serious mental illnesses who are sexually active may be more likely to engage in high-risk behaviours, including inconsistent condom use, having multiple sexual partners, engaging in transactional sex and consuming alcohol before sex, which may increase the likelihood of unprotected sex.^16^,^18^ Additionally, IPV can precipitate mental health challenges such as post-traumatic stress disorder (PTSD), anxiety, depression, suicidal thoughts and substance use disorder.^19^,^23^ Studies have shown that women experiencing IPV are at higher risk of developing these mental health challenges due to the chronic stress and trauma associated with ongoing abuse, as well as the possible isolation from social support systems.^24^,^26^ Conversely, poor mental health can make individuals more susceptible to IPV due to factors such as low self-esteem, emotional dependency and difficulty recognising abusive behaviours.^27 28^ Moreover, the relationship between IPV and HIV involves both biological and sociocultural pathways.^29^ Biologically, forced or coercive sexual intercourse with an HIV-infected partner can lead to HIV and other sexually transmitted infections.^30^ Socioculturally, violence and the fear of violence compromise the women’s ability to negotiate condom use, which can lead to inconsistent condom use and thereby increase HIV risk.^31^

An important aspect of syndemic theory is that structural and environmental factors impact the disease clustering and often exacerbate the syndemic conditions.^4 32^ These include poverty, gender inequality, stigma and lack of access to resources and services that could improve the health and well-being of individuals.^33 34^ Financial stress and lack of resources can exacerbate mental health challenges, due to the higher levels of stress as a result of financial insecurity, job instability and poor living conditions, which are significant risk factors for depression and anxiety.^35^ Furthermore, financial dependence on a partner can significantly reduce one’s bargaining power within the household making it difficult to leave an abusive situation.^36^ In addition, people living in poverty may engage in risky sexual behaviours to meet their basic needs, which can elevate their risk of contracting HIV.^37 38^ Cultural factors, such as gender norms and expectations, also influence the epidemics.^39^,^41^ For instance in many societies, traditional gender roles can reinforce power imbalances, making women more vulnerable to IPV and less able to negotiate safe sex practices, increasing their risk of HIV.^41^ Additionally, stigma associated with IPV, poor mental health and HIV can hinder individuals’ willingness to seek help and adhere to treatment leading to further isolation, untreated conditions and exacerbation of both physical and mental health challenges.^42^,^44^

Other factors, such as adverse childhood experiences, can also increase the risk of the co-occurrence of the epidemics. Additionally, adverse maternal experiences in childhood not only elevate the risk of poor mental health in adulthood but also increase the likelihood of experiencing IPV and acquiring HIV,^26 45^ which, as previously noted, significantly impact parenting practices. Child sexual abuse is alarmingly prevalent in South Africa.^46^ A nationally representative cross-sectional study among 5631 households found that 9.99% of boys and 14.61% of girls reported experiencing sexual violence in their lifetime, with physical abuse, emotional abuse and neglect strongly associated with sexual violence.^46^ Trauma from child abuse is a significant factor contributing to adult mental health challenges such as PTSD, depression and anxiety.^26 45 46^ The mental health challenges as a result of the abuse may increase the survivors’ risk of being involved in violent relationships and as a result, elevate their HIV risk.^26 47^ Survivors of abuse often struggle with establishing healthy relationships due to the trauma, which can lead to low self-esteem, emotional dependency and difficulty in setting boundaries.^26^ This dynamic, combined with the impaired ability to negotiate safe sex, significantly heightens the risk of HIV infection among these individuals.^47^

### Parenting practices among women living with HIV, IPV and poor mental health syndemic

Parenting is influenced by various factors, including the characteristics and experiences of parents and the context in which they are raising their children.^48^ Mothers living with HIV encounter unique challenges.^49 50^ Some evidence indicates a heightened risk of psychiatric vulnerability among women with HIV, especially in sub-Saharan Africa,^51^ which is exacerbated by disease-related stress and structural challenges such as poverty,^52 53^ gender inequity^51^ and limited mental health services.^54^ Maternal HIV can negatively impact both infected and uninfected children’s developmental, psychological and physiological well-being.^55^ These adverse outcomes arise partly from compromised parenting due to maternal stress and mental health deterioration, potentially leading to neglect or aggression towards the child.^56^ This is especially significant in sub-Saharan Africa, where 1.36 million pregnant women were living with HIV as of 2010.^57^

IPV further complicates the parenting landscape. High rates of IPV among heterosexual couples indicate that many children are raised in violent households.^58^ Early exposure to IPV negatively impacts children’s mental health, resulting in trauma, depression and anxiety.^59 60^ A meta-analysis of 118 studies revealed a 50%–63% likelihood of children developing internalising problems and clinical disorders due to violence exposure.^61^ IPV impacts children’s emotional regulation and introduces environmental stressors that may result in mood swings and low self-esteem among the children, thereby making the parenting experience more difficult.^62 63^ Additionally, mothers experiencing IPV are more likely to resort to harsh discipline, sometimes escalating to physical abuse, both to placate their abusers and as a result of displaced aggression.^64^ The emotional toll, often manifesting as depression, affects the quality of mother-child interactions and can erode mothers’ confidence in their parenting capabilities.^65 66^

Poor mental health adds another layer of complexity to parenting. Parental depression is significantly associated with more hostile and harsh parenting.^67 68^ With 10%–20% of women experiencing depression in their lifetime and more than 30% having persistent depressive symptoms.^69 70^

It must be noted that although HIV, IPV and poor mental health can lead to harsh parenting, there are protective factors that can buffer these socioemotional outcomes. Resilience, social protection and the ability of some mothers to act as emotional anchors during moments of violence or distress can mitigate the impact of IPV.^71 72^ For mothers living with HIV and poor mental health, access to antiretroviral therapy, social support and effective mental health services can improve parenting quality and mitigate negative impacts on children.^73 74^

### Cultural significance of parenting in South Africa

Parenting practices in South Africa are deeply influenced by cultural norms and values.^75^ The extended family often plays a crucial role in parenting, with grandparents, aunts, uncles and older siblings actively involved in the upbringing of children.^75^,^77^ According to Statistics South Africa (2023), households with more than four children are more likely to have children cared for by extended family members (84.5%). Furthermore, the same survey found that a fifth of all children did not live with both their parents, and most children lived with only their mothers and other extended family members (42.0%).^77^ This collective approach to parenting is rooted in the African concept of ‘Ubuntu’, which emphasises compassion for others and the interconnectedness of all people.^78^ Traditional gender roles also influence parenting, with women generally seen as the primary caregivers responsible for day-to-day nurturing and education of children, while men provide financial support.^76^ Fragmented families in South Africa can be attributed to labour migration and low marriage rates especially among African women, who are also less likely to live with the child’s father if they are not married.^76 79^ The historical and socio-political context of South Africa, particularly the legacy of apartheid and ongoing socioeconomic challenges, significantly shapes parenting practices.^80^ The psychological impact of the apartheid-era trauma has affected parenting as parents who experienced trauma may struggle with emotional regulation, influencing their ability to engage in consistent and nurturing practices.^80^ Additionally, during colonialism, corporal punishment was institutionalised as a method of maintaining order and discipline in both schools and homes.^81^ As a result, many South African homes today still view corporal punishment as integral to teaching children respect for authority and obedience.^81 82^ Despite the legislative ban on corporal punishment, many mothers continue to practice this form of parenting.^83^ A qualitative study found that parents disagreed with the law and held positive attitudes towards corporal punishment, viewing it as effective for disciplining children.^82^ Mothers often used corporal punishment to correct children’s behaviour, lacking knowledge of alternative disciplinary methods.^82^ The use of corporal punishment is deeply embedded in cultural and historical contexts, passed down through generations as a normative practice.^82^

### Present study

The current study aims to explore parenting among women living with the different syndemics (specifically, HIV-Mental Health; IPV-Mental Health; and HIV-Mental Health-IPV) and how these syndemic conditions influence parenting practices. This research moves beyond examining each syndemic in isolation to highlight how their inter-related and compounded effects further complicate parenting. In a context where millions of children and mothers grapple with these complex epidemics, a nuanced understanding of these interactions offers valuable points for targeted interventions.

## Methods

### Study design

The study employed a qualitative study design using a narrative approach. A narrative approach focusses on exploring the stories of individual’s experiences as narrated by the participants themselves in their unique contexts and environment.^84^ The basis of the approach is that people’s narratives provide a rich and nuanced insight into their life experiences.^84^ The use of qualitative methods allowed for a comprehensive exploration of how participants, living with the intersecting epidemics of HIV, IPV and poor mental health, approached parenting and how these epidemics influenced their parenting experiences.

### Study setting

We conducted the study in two sites within Mpumalanga Province, South Africa, from October 2022 to February 2023. In 2022, Mpumalanga Province reported an estimated population of approximately 4 720 497 people.^85^ The primary languages spoken include siSwati (27.67%), isiZulu (24.1%), Xitsonga (10.4%) and isiNdebele (10%).^86^ In 2019 there were 1.26 million people living in poverty across the district,^87^ with high levels of unemployment at 36.69%.^87^ Recent data based on a population-based household survey indicated an HIV prevalence of 20.8% among adults aged 15 years and above,^88^ with approximately 38% physical IPV prevalence among girls and young women in Mpumalanga.^89^ Furthermore, data analysis conducted on a sample of 4351 adult South Africans from various ethnic groups indicated a lifetime prevalence estimate of all common mental disorders, with Mpumalanga at 29.2%.^90^ Additionally, the study location has a notable lack of mental health services, with only one clinic offering counselling services and one psychologist available within this facility.

### Sample

A purposive sampling approach was used to recruit 20 women between the ages of 20 and 60 years, using specific survey items from the Interrupt_Violence Study as the screening criteria.^91^ These survey items assessed participants’ experiences with HIV, poor mental health and IPV. The inclusion criteria required the women to have experiences with at least two of the three epidemics: IPV, poor mental health or HIV. The experiences of IPV could be current or past, while poor mental health needs to be current. Poor mental health was assessed using several tools: the 4-item Ask Suicide Screening Questions,^92^ the 7-item Generalised Anxiety Disorder Questionnaire^93^; the 9-item Patient Health Questionnaire for depression^94^ and the 8-item Post Traumatic Stress Disorder Questionnaire.^95^ For IPV, participants had to respond ‘yes’ to any of the questions in the WHO instrument^96^ for physical, emotional and sexual violence victimisation experiences. HIV status was either self-reported by the participant or assessed through voluntary HIV testing conducted by the fieldworker. Additionally, participants had to self-identify as caregivers for a child or children under the age of 17. This was determined by asking participants in the survey, ‘For how many children are you a caregiver for like a parent?’. Participants who answered more than zero were further asked about their relationship with the child, with common responses being mother, aunt or grandmother. Men were not included in the study because it is usually women who are the primary caregivers for children in South Africa and who are disproportionately affected by the epidemics under investigation.

### Patient and public involvement

The research question and aims of the current study were informed by the priorities, experiences of the participants in the community. The findings from the main study, which was a longitudinal study found that the adolescents that were initially interviewed had experiences of violence and subsequent mental health challenges. These findings informed the follow-up research question, which sought to explore how these past experiences impacted them in the present. However, participants were not involved in the design of the study or the recruitment process. Results were disseminated through the community advisory board, where members actively engaged in discussions on how the findings could be used to resolve community challenges.

### Data collection tools and procedure

Through in-depth open-ended interviews using an interview guide comprising 20 questions, we were able to elicit detailed narratives from the participants, capturing their perspectives, emotions around parenting. All the interviews were conducted face-to-face by two research assistants and a PhD candidate, either in the participants’ homes or at a location of their choice, with each interview lasting 60–90 min. The local languages of Xitsonga and SiSwati were used during the interviews which were audio recorded, translated and transcribed verbatim by trained transcribers. The translation process was rigorous to ensure the accuracy and cultural relevance of the transcripts with bilingual transcribers, fluent in both the local languages and English, who were also responsible for the translations. In instances where there was no equivalent word in English, the transcribers included the siSwati or Xitsonga word and provided an explanation in English to maintain the meaning of cultural phrases. Additionally, the research team reviewed the translated transcripts alongside the original audio recordings to verify the accuracy of the translations.

Participants were given a ZAR50 ($271) grocery store gift card as compensation for their participation in the study. This amount was chosen as it is not deemed to be coercive in the setting (rural and peri-urban Mpumalanga). Each interview explored childhood experiences of the participants which included experiences of childhood abuse, relationships with mothers and women caregivers and their intimate relationship dynamics. Critical to the study was also the participants’ relationship with their children, their feelings about being a parent and any challenges related to parenting.

The in-depth interviews were supported through the use of arts-based methods. The interviewers were trained in the arts-based methods by one of the coinvestigators, a therapist and researcher with expertise in psychology, play therapy and art therapy. The arts-based methods were used to stimulate and encourage dialogue among the participants. Participants were presented with three activity options: Kinetic Family Drawing (KFD), River of Life and Sandbox. The KFD is a method that taps into participants’ memories and childhood experiences.^97^ It is also useful for providing insight into perceptions of family dynamics.^98^ In relation to the KFD, participants engaged in two drawing activities: one representing their family of origin and another depicting their current family engaged in an activity. They explained the content of both drawings to the interviewer using open-ended questions.^97^ The River of Life method facilitated participants’ reflection on their life events, as they drew a road symbolising their life journey and marked significant events.^99^ The sandboxing method which drew from sand play therapy, involved participants creating three-dimensional scenes in a tray filled with sand using various figures. They were encouraged to freely select figures and construct their own world in the sand, expressing their thoughts and interpretations.^100 101^ Each figure held personal significance for the participants, serving as a tool for deeper communication.^100 102^

Data saturation was assessed through the iterative process of data collection and analysis. This iterative process involved collecting and analysing the data in cycles. After each round of data collection, the data was immediately reviewed and analysed to identify emerging themes and patterns. Based on this preliminary analysis, subsequent data collection efforts were adjusted to explore the themes in greater depth or to investigate any new areas that may arise.

After each round of interviews, the research team reviewed the findings to identify recurring themes and patterns. As data collection progressed, we observed that no new themes or insights were emerging, and the information provided by participants began to repeat. This repetition, or redundancy, indicated that we were not gaining any new insights from additional data, suggesting that data saturation had been reached.

Moreover, the consistency of the themes across multiple interviews reinforced our confidence that we had comprehensively captured the participants’ experiences. While formal discussions on data saturation were not explicitly conducted among the study team, the iterative nature of our data collection and analysis provided a clear, practical indication that data saturation had been achieved.

### Research trustworthiness

To ensure the quality of the research we adhered to the criteria of credibility, transferability, dependability and confirmability.^103^ Credibility was ensured through deep engagement with the local community. Our qualitative interviewers spent 5 months in the community, engaging with the participants to build trust and gather rich data. Prior to the qualitative interviews, the main study and quantitative interviewers had also established rapport with the participants, having been in the community for 2 years. The research team was highly qualified and interdisciplinary, including a social worker, a trauma therapist and experts on violence against children (VAC), IPV research and community engagement. To support peer debriefing, the management team held weekly sessions with the interviewers. These sessions allowed the team to discuss ongoing interviews, strategise and address any challenges related to data collection and provide emotional support to the interviewers. To ensure dependability, a detailed proposal was developed outlining the qualitative data collection process, and the interviewers maintained thorough field notes. Intercoder agreement and reliability were ensured through team reviews of the initial six transcripts, guaranteeing consistent application of the codes.

To ensure confirmability, reflexivity played a critical role. Weekly debriefing meetings served as a platform for the research team to reflect on the research process, discuss any biases and examine how our backgrounds might influence data collection and interpretation. Field notes were taken following each interview to assist in the research process, serving as a record of the researcher’s thoughts and reactions. These notes facilitated ongoing reflection on how the researcher’s role might influence the study’s findings and interpretations. Furthermore, each team member continually reflected on their position. The research team comprised individuals with diverse backgrounds, including two black women with postgraduate qualifications (one Master’s student and a PhD candidate), one black man who was a Masters student, and three white women with PhD qualifications. The researchers acknowledged their middle-class status and postgraduate qualifications while working in these communities marked by severe poverty. This self-awareness was essential to understand how our socioeconomic status, race, gender and educational background could impact our interactions with participants and the data collected. For instance, being interviewed by a man may have influenced the willingness of some of the female participants to disclose certain experiences. By actively reflecting on these factors during the weekly debriefing meetings and throughout the research process, the team aimed to mitigate potential biases and enhance confirmability of the study’s findings. To ensure transferability, we provided detailed descriptions of the study context and participant characteristics, allowing readers to understand whether the findings might apply to their own settings.

### Data management and analysis

A typology of participants was developed deductively using the syndemic theory. A broad coding framework was developed based on the research objectives and an initial thematic review of selected transcripts.^104^ This framework was informed by key concepts from syndemic theory, such as the interactions between IPV, mental health and HIV and the contextual factors that lead to the clustering of the epidemics, and served as a guide for coding the data.^34^ We then identified inductive codes based on a deep reading of the transcripts and by comparing and contrasting the findings between the categories of participants based on the syndemic theory.

Thematic analysis, following the six-step process outlined by Braun and Clarke, was used to analyse the interviews.^105^ The steps included familiarising ourselves with the data, searching for emerging themes by examining the coded data to identify broader patterns of meaning, reviewing themes to ensure that they accurately reflected the data, and applying the themes to the data during the write-up.^106^

## Results

### Characteristics of participants

The study included 20 participants, between the ages of 22–60, with a mean age of 36 years. Seven participants experienced the co-occurrence of all three epidemics (HIV-Mental Health-IPV). Their ages ranged between 24 and 45 years, and they were caring for one to four children. Eight participants were living with the HIV-Mental Health syndemic. This group was more varied in age, spanning from 23 to 57 years, with caring responsibilities ranging from one to five children. Five participants were living with the IPV-Mental Health syndemic. These women were between 22 and 49 years old and were caring for two to five children. Across all the groups, participants reported symptoms related to poor mental health. However, only one participant had accessed mental health services at the provincial hospital, while the others had not accessed any formal mental health support before. Those that were living with HIV had received only HIV-related counselling from the clinic which mostly covered their status disclosure to loved ones and adhering to treatment, but they did not receive any counselling related to specific mental health challenges. HIV-positive participants reported living with HIV for durations ranging from 2 to 18 years.

#### Summary of key themes

**Figure 1:**
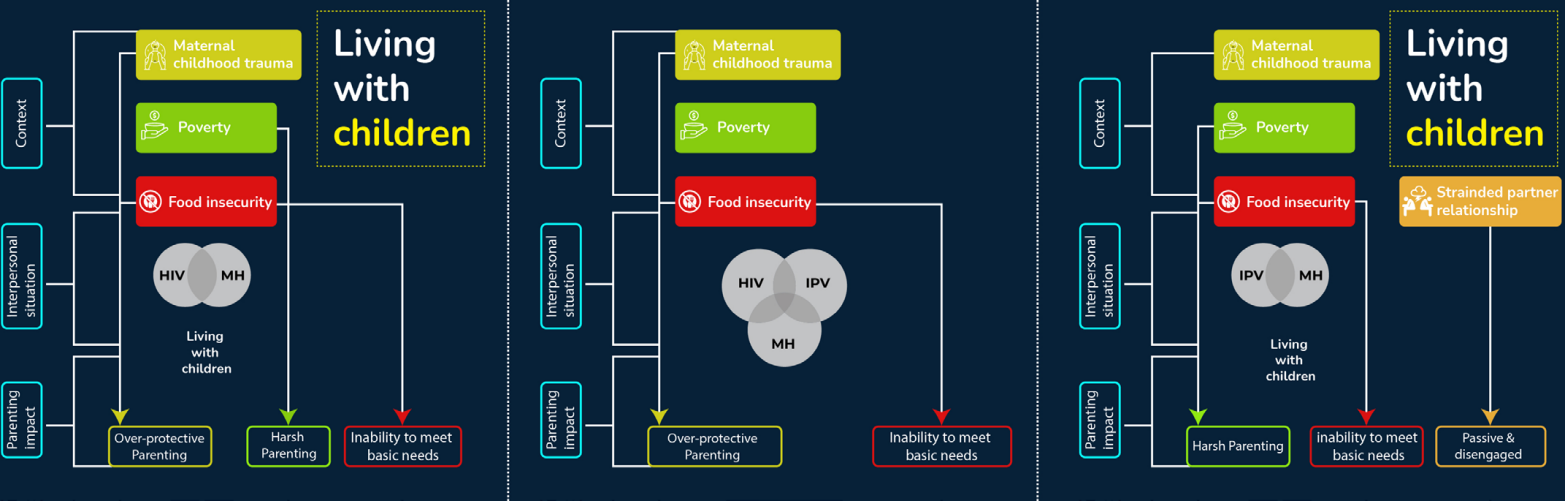
The syndemic and the underlying factors influencing specific parenting outcomes (HIV-Mental Health, HIV-IPV-Mental Health, IPV-Mental Health syndemic from left to right). IPV, intimate partner violence.

The study identified several factors among women experiencing the syndemic conditions that had an influence on their parenting practices. The four themes generated included mothers being separated from their children due to adverse circumstances, strained relationships with the fathers of their children, parental experiences of childhood trauma, including neglect, physical and sexual abuse, and significant socioeconomic challenges that hindered their capacity to meet the basic needs of their children. Figure 1 provides a graphic representation of the findings.

### Separated by adverse circumstances: the influence of living arrangements on parenting practices

Participants who had experienced all three concurrent epidemics of HIV-Mental Health-IPV reported being separated from their children. In most cases, their children were living with the participant’s mother or aunts. However, participants’ reported seeing their children on most weekends. Various reasons were cited for why they did not live with their children, some described financial constraints related to looking after the children, while others described how family members insisted on raising the child because the participant was an adolescent when the baby was born and viewed as too young to look after the child, and in some cases family members feared for safety of the child, especially if the mother was experiencing IPV. One of the participants, Dixy Rose, in her 20s, specifically pointed to financial constraints such as the costs associated with school transportation as one of the reasons, she was not able to live with her children. Despite her reservations about how her children were treated by her mother Dixy Rose felt she had no other choice, she remarked,

When the schools are open, I send my children to my mother because the school is closer to her, and I can’t afford to pay for transport. However, I fear that she doesn’t treat them the same way I do. They tell me she doesn’t treat them well and hits them when they are naughty. But what can I do? They need to go to school.

Furthermore, safety considerations also played a role in participants’ decision-making, Dixy Rose also disclosed how her family had concerns about the children’s safety, she further explained,

My grandmother also convinced me not to take my children because we live in a one-room place. She said I would end up having sex in front of the kids, which is something I would never do. We would never do that. She was also afraid that the man I am staying with now might rape my children, as it’s unfortunately common for men to do so lately.

Despite participants in the HIV-Mental Health-IPV group not living with their children, they often described their relationship with them in loving and warm terms expressing that they loved being a parent and believed that they had a good relationship with their children. When asked about her relationship with her children Dixy Rose shared,

When my children are around, I am very happy. I have conversations with them, we chat like friends, and I play with them.

Sage, also within the same age range, shared similar sentiments, expressing the following,

I just wish I lived with my children so we could be happy together. I want to raise them and guide them according to my own beliefs. I’d love for them to have what I didn’t have as a child.

Moreover, none of the mothers and caregivers within this group reported behaviours that could be interpreted as child abuse and stated adamantly that they did not engage in corporal punishment. Phoenix, in her 20s, with two children had the following to say in relation to how she disciplined her children*,*

I decided and told myself that I do not want to be a bully to them. I do not want them to be scared whenever they see me. I do not want to make them live that kind of life. I can’t hit them because I love them so much.

Similarly, Sage also revealed, ‘I sit them down and talk to them. I feel like hitting children makes them worse’.

This was notably different from the women from the HIV-Mental Health and IPV-Mental Health groups who were living with their children, as they all reported using corporal punishment to discipline their children. Halo, who was in her 40s from the IPV-Mental Health group, openly shared how she disciplined her children,

I hit my children with a stick. I hit them when they misbehave. I want them to listen. I don’t like children that are disrespectful.

Similarly, when Phoenix was asked how she guides her children, she reported ‘I hit her and sometimes I reprimand her, it depends’.

Additionally, mothers living with the HIV-IPV-Mental Health syndemic, who were not living with their children, expressed profound fears for their children’s safety. Despite family members stating that such living arrangements were in the best interests of both the mother and child, these mothers had concerns that their children might be exposed to traumatic experiences similar to what they themselves had endured, such as child sexual abuse. Avatar in her 30s shared her fears about her children,

I try by all means to show my children love and to protect them from the hardships and struggles I went through. I was raped and grew up in a situation where, when my grandfather was drunk, he would become violent. We knew that when he was drunk, we needed to find a place to hide to protect ourselves. At least my children are protected because I do everything I can to ensure they are safe and have food to eat.

Sage further reported, ‘I don’t want them to grow up with that anger that I grew up with, and to see the things that I have seen. I’d love for them to get what I didn’t have as a child’.

### Strained relationships with fathers of the children and their influence on parenting practices

Participants from the IPV-Mental Health group had strained relationships with the fathers of their children. In most cases, we found that the father of the children was usually the perpetrator of IPV. Halo’s (40–50 years) experience highlights the severity of this issue, ‘That one is very violent. He is very violent. When I was really sick in 2020, he would beat me badly. He would come and slap me for no reason’. Quinn (HIV-Mental Health-IPV), also within the same age range had a similar experience with the father of her children being abusive towards her, she recalled,

When I was 22, I had my second child. Between the ages of 25 and 27, I had my second boy. However, my husband drank a lot, smoked cigarettes, and used marijuana, which led to him abusing me. When it was just the two of us, he wasn’t abusive, but after we had children, he started abusing me. This continued until I had my last child, who sadly passed away. He drank heavily and abused me until I was about 35 years old.

Participants in this group also shared that the fathers of the children did not support the children whether financially or emotionally. Phoenix, who was in her 20s, shared that the father of her children did not play an active role in the children’s life, Phoenix disclosed,

No, he doesn’t discipline her, he doesn’t do anything. I: Does he support your child financially? P: No, but he comes and sees her. He was here in December to see her. He sees her about once a year.

Conversely, participants from the HIV-Mental Health group reported having a good relationship with the fathers of their children, even if they were no longer romantically involved in some cases. Participants reported that the fathers of their children supported and were involved in the children’s lives. Star, who was in her 20s, shared the following about the father of her child,

The biological father is around, I’m not even in a relationship with him anymore but he is still very involved in PR’s life. He picks her up and they still do things together.

Aspen, who was also in her 20s, also expressed a similar sentiment, that despite no longer being in a romantic relationship with the father of her child, he was still involved in their son’s life,

Since we were against getting an abortion, the family saw us as disrespectful, which strained our relationship. As a result, my relationship with him didn’t last. Whenever he wanted to see me, he couldn’t. However, he remains involved in our son’s life and supports him financially. My child visits him whenever he wants, and they have a great relationship despite us no longer being together.

When it came to parenting in both the HIV-Mental Health and IPV-Mental Health groups, we found that participants often used corporal punishment as a means of disciplining their children. Participants in the IPV-Mental health group also shared that their experiences of IPV affected how they disciplined their children. Despite both groups using corporal punishment as a means of discipline, there was a difference in how they described their relationship with the children. The mothers and caregivers from the IPV-Mental Health group reported an inability to connect with their children and a sense of detachment and dissatisfaction with parenting, Phoenix (IPV-Mental Health) shared her dissatisfaction with being a parent, stating,

I wouldn’t want to be a parent if I had a choice. Nothing makes me happy about being a parent [laughs]. To be honest, I just don’t find joy in being a parent.

Phoenix (IPV-Mental Health) further described how her abusive relationship affected her interactions with her son:

When I was in that abusive relationship, my son would try and speak to me, I would just insult him. I would get pissed off by my partner and take it out on the closest person to me and usually, it was my son he would do a small thing, like spill water, and I would overreact. I realised that the way I was hitting him was not normal. I would take out a lot of pain on him.

On the other hand, the caregivers who had experienced HIV-Mental Health described a closer connection with their children and a more positive overall relationship. This group, in some cases, even referred to their children as their friends. Bailey, who was in her 20s, shared, how despite initially having some difficulty with parenting, she loved her daughter,

She is a blessing, I didn’t want to have a baby, but now that I have her, she has made my life better. It’s just that we started off on the wrong foot, but we are great now. I am happy to be her mom. She is my friend, but we have to maintain the respect that I am her mother, and she is my daughter.

Furthermore, although the mothers and caregivers in the HIV-Mental Health group expressed a close relationship with their children, this did not stop them from using corporal punishment as a means of disciplining their children. When Aspen was asked how she disciplined her children she responded,

I scold them or hit them with a tree branch. I hit them when they are naughty. I hit them with a stick.

Caregivers in this group justified corporal punishment as an important strategy in guiding their children to do the right thing or ensuring that they comply or behave, Aspen further explained when talking about her son,

I usually hit my oldest child because he’s naughty and likes hitting other kids. Before I hit him, I scold him several times and give him chances to do the right thing. But if he continues to misbehave, I get angry and then hit him.

### Parental experiences of childhood trauma and their influence on parenting practices

We found that participants from all the syndemic groups had challenging childhood experiences. More specifically all the women reported having experienced physical abuse from their caregivers which mostly included being hit with a stick as a form of discipline. Milan (HIV-Mental Health) expressed how her mother would hit them even when they had not done anything wrong,

She was the type of person who would beat you up for having a headache under the assumption that you are lying and just lazy.

Aspen recalled similar experiences from her childhood ‘She would hit me with a belt or a tree branch. Whenever she asked me to do something, she expected me to drop everything I was busy with and attend to what she wanted me to do’.

The women from the HIV-Mental Health and HIV-IPV-Mental Health groups both reported that they had experienced exposure to parental violence. Avatar (HIV-Mental Health) shared,

Whenever my grandfather drank alcohol, whether monthly or when he had just been paid, he would always hit her. He would even chase her to the neighbours’ house. It was hard, but we knew what to expect whenever he was drinking. This went on for a long time. At one point, he even hit me on the back of my head, and I had to get stitches.

The women living with the HIV-Mental Health syndemic were the only ones who explicitly reported emotional and physical neglect from their caregivers. Within this group, participants recounted instances where they were denied access to education because they were required to care for younger siblings and in some cases, they were deprived of food as a means of discipline. Milan (HIV-Mental Health) shared,

I was not allowed to go to school, and when the neighbours asked my mother why she didn’t send me, she would reply by asking who would look after the other kids she had later. She had another child, and I had to take care of that one as well. So, I stayed at home while my peers went to school.

Childhood sexual violence was only reported among women living with the HIV-IPV-Mental Health syndemic. These participants shared accounts of sexual violence, starting from a young age, perpetrated by relatives or close family friends, Phoenix recalled some of her traumatic childhood experiences,

I was once raped, not once but three or four times, if I’m not mistaken. At that time, I was 9 years old. My life was like that. I grew up with this thing, it lives in me. I never got the chance to go for counselling. This guy was a family friend, he used to come to my parents’ place.

I remember well, at that time, they were selling beers at my grandmother’s place. On the day it happened, he decided to do what he wanted. He took me for the whole day. Then he did what he wanted to do. He raped me. He said that if I told someone about it, it would be the end of me.

Additionally, some participants in this group described instances of disbelief from their caregivers when they disclosed the abuse they endured. This absence of support led to feelings of confusion, depression and anxiety. Sage (HIV-IPV-Mental Health) shared,

I didn’t know anything about sex, I knew nothing and he raped me. When I went back to my aunt’s house, I told them what happened. I explained to them what he did to me because I didn’t really understand it and I needed them to tell me what had happened to me but they said I was lying. When they accused me of lying, I started thinking that maybe what had happened was actually my fault and that there was something that I had done wrong. The situation was not good at all and then I started having serious mental health issues.

When it came to parenting, a pattern among these participants was their overwhelming fear and concern for their own children. Their primary worry was that their children would endure the same traumatic experiences they had faced. As a result, participants displayed signs of overprotectiveness and an intense fear that harm might befall their children. However, this sense of overprotectiveness was more common in the HIV-Mental Health-IPV and HIV-Mental Health groups. Some caregivers even admitted to being hesitant to allow their children to leave the home to play with other children due to their apprehensions about potential dangers.

Dixy Rose (HIV-Mental Health-IPV) expressed,

I don’t want my children going out to play with the neighbours’ children or being out and about. I prefer for them to be at home. As you can see, every time you have been here, my children are around me. I don’t want my children to grow up the way that I grew up. I don’t want them to see the things that I have seen.

Similarly, Aspen, also shared her fears regarding the children’s safety, ‘If it were up to me, they wouldn’t play where I can’t see them. Even when I go to town, I call them constantly to check that they are alright. I don’t feel comfortable when they are out of sight. It scares me’.

### Socioeconomic challenges and their influence on parenting practices

Most of the participants from all the syndemic groups faced an interconnected web of socioeconomic challenges, beginning with early life traumas such as sexual violence, parental loss or physical abuse. These traumas often led to early school dropouts, setting the stage for ongoing struggles with unemployment, poverty and food insecurity. Quinn (HIV-Mental Health-IPV), who was in the 40s reflecting on her past, shared,

We were very poor when I was growing up. My grandmother couldn’t afford to keep sending me to school, so I had to leave. Then I found a partner and became pregnant. Growing up was tough, very tough, because my mom was the only one working and she had to look after all of us. She had to do everything for us—feed us, make sure we had clothes for school, and so on. So, it was tough. When I was 15, I was no longer living at home. I fell pregnant with L, couldn’t go to school, so I just got married and had a baby. But this man was very abusive and drank a lot, so we really struggled.

The sequence of these challenges subsequently influenced their capabilities as parents. For women living with the HIV-Mental Health-IPV syndemic as mentioned earlier their socioeconomic challenges posed obstacles to taking full custody of their children. Participants across all groups highlighted their difficulty in meeting the basic needs of their children due to these persistent socioeconomic hardships. Common issues included reliance on neighbours or relatives for food staples such as maize meal, soap as well as limited scope for recreational activities with their children. Furthermore, participants reported that they relied on receiving social welfare grants from the government. These grants are means-tested and provide financial assistance to no or low-income families and include the Child Support Grant (ZAR 510/$27.77); the Older Person’s Grant, which supports individuals over 60 years of age (ZAR 2090/$113.79); the Disability Grant, which assists those aged 18–59 with disabilities (ZAR 2090/$113.79); and the social relief of distress grant, which offers 3-month temporary aid for those in severe financial hardship (ZAR 350)/$19.06).^107^ While these grants provided some financial relief, they were not always consistently paid.

Indigo (HIV-Mental Health-IPV) who was in her 40s reflected,

My children and I rely on the grant for our livelihood, and we live here on this plot. I wish I had more money to fix the home we live in and to buy a Jojo tank because our biggest problem here is water. I just wish we always had water.

Beyonce (HIV-Mental Health), who was in her 40s, expressed how she also had a difficult time making ends meet and relied heavily on the support of her neighbours,

Most of the time, my neighbour shares maize meal with me because sometimes, like today, the 15th, we still haven’t received our R350 social relief of distress grant. Even when we do get the grant money, it’s often not the full amount, so it’s not enough for food. I also have to buy other necessities like soap and toothpaste. Sometimes, the soap runs out in the middle of the month. Just like now, we are not going to get the money anytime soon, and a friend gave me this soap after hearing me say we had none. Another friend gave me R20, and I bought toothpaste because we didn’t have any.

Some participants expressed that the only leisure time spent with their children was when they could afford it after receiving government grants often culminating in rare trips to the mall. Dakota (HIV-Mental Health), who was in her 20s, expressed the limitations of her financial situation on what she was able to do with her child,

No, I’m not working; I’m just sitting at home. I survive on his child grant, which comes at the end of the month, and then I can take my son to the mall.

Furthermore, the cumulative effect of these socioeconomic challenges manifested in heightened levels of stress and reduced self-efficacy in parenting roles across all participant groups.

Beyonce (HIV-Mental Health), when thinking about her capabilities as a parent shared:

I do not see myself as a good parent, like those who can take proper care of their children. I do not know how to, and I have not been able to do it. Because I did not work, I do not have anything to offer them. I cannot do all the things they want me to do for them.

Additionally, Dakota (HIV-Mental Health) also shared her parenting challenges,

The most difficult thing about parenting, I think, is not having money because a child has so many needs, and it is difficult to meet all of them. He needs things for school, and sometimes I have to send him to school without food, so we have to ask the neighbours for help*.*

## Discussion

Our findings revealed that the syndemic interplay of HIV, poor mental health and IPV places a compounded burden on parenting outcomes. Moreover, we found that the experience of the different syndemics was associated with certain contextual factors which influenced the way the caregivers parented. Specifically, parents living with the three epidemics (HIV-Mental Health-IPV) had been separated from their children due to the family’s perception that they were unable to adequately parent them. Those grappling with a dual burden of IPV-Mental Health, while living with their children, experienced strained relationships with the fathers of their children and exhibited disconnection and emotional distance toward their children. Across all groups—whether they were living with the full HIV-Mental Health-IPV syndemic or other combinations of these, HIV-Mental Health or IPV-MH, childhood trauma and socioeconomic challenges emerged as influential factors that adversely affected their parenting practices.

### Separated by adverse circumstances: the impact of living arrangements on parenting practices

Our research findings highlight the compounding influence of the HIV-Mental Health-IPV syndemic on mothers, making it notably more challenging for them to live with and care for their children. Family members often perceived these mothers as unfit for caregiving and as a result removed the children from their care. This assessment of the mothers being incapable was far less common for mothers living with just two of these epidemics (HIV-Mental Health or IPV-Mental Health). The removal of children from their care also highlights the harsh societal judgement and stigma faced by mothers at the intersection of these three stigmatised epidemics. In the context of South Africa, where high HIV mortality rates among young people are prevalent, it is not unusual for other family members like grandparents to step in and assume caregiving roles.^108^ Such living arrangements relieved these mothers of some immediate caregiving responsibilities and we also found that they also led to the complete avoidance of corporal punishment as a disciplinary method. Multiple explanations could exist for this, first this could be due to the variable levels of social support available to mothers not living with their children.^109 110^ When caregivers and mothers perceive a sense of support from their social circles, they are often inclined to use positive parenting behaviours such as showing affection, supervision and being more supportive, even when they are dealing with substantial difficulties or obstacles.^111^ Another explanation could be the emotional well-being of the mothers, it could be that the separation from their children led to heightened emotional states like guilt or shame, potentially influencing their preference for less aggressive disciplinary methods. Finally, a third explanation could be the possible abdication of parenting responsibility, which may potentially stem from internalised stigma concerning their parenting capabilities or confidence in their parental role. The latter could potentially have led them to shy away from disciplinary responsibilities that were commonly used in the community.

Despite the legal prohibitions against corporal punishment, its continued prevalence in South African homes and schools reflects the enduring societal norms that fail to recognise it as a form of violence.^112 113^ The cultural acceptance of corporal punishment in peri-urban Mpumalanga could also shed some light on why mothers from the current study, specifically from the IPV-Mental Health and Mental Health-HIV groups, continued to use corporal punishment as a disciplinary method. As noted, this was in contrast to the women living with the HIV-IPV-Mental Health syndemic, who did not live with their children. Taking this context into consideration, it is reasonable to assume that the mothers from the IPV-Mental Health and HIV-Mental Health groups who did live with their children, would adopt disciplinary methods common within the broader community.

It is worth noting how most existing studies on the effects of parent-child separation tend to focus on the non-custodial parent, often the father,^114^ or on the perspectives of primary caregivers like grandparents.^115 116^ Our study fills a significant gap by concentrating on how the syndemic of HIV-Mental Health-IPV specifically influences mothers’ relationships with their children when they are not cohabiting, thereby offering unique and critical insights into this complex issue.

### Strained parent relationships and the influence on parenting practices

Living with IPV-Mental Health complicates parenting. Our research found that mothers with this combination often had strained relationships with the fathers of their children, which exacerbated existing Mental Health challenges such as stress, anxiety and depression. This strained relationship further reinforced harsh parenting behaviours, such as disengagement and passivity.^117^ According to Belsky’s ecological model and the family systems theory, the quality of the romantic relationship influences the parent-child dynamic, a phenomenon known as the ‘spillover effect’, where the increased conflict, mistrust and discomfort in closeness caused by IPV can in turn can also shape the nature of the parent-child relationship in the same way.^118^ Stress and other mental health challenges related to IPV further undermine a mother’s ability to parent positively. This deterioration in maternal mental health can manifest as reduced patience, less effective communication and a decreased ability to manage the daily responsibilities of caregiving, ultimately affecting the parent-child relationship in a detrimental way.^119 120^ A systematic review by Chiesa *et al* support this, revealing that women facing higher levels of IPV engage less in positive parenting practices including sensitivity, engagement and expressing positive emotions.

Conversely, mothers with the HIV-Mental Health syndemic who maintained healthier relationships with the children’s fathers showed more nurturing behaviours towards their children. This finding aligns with research by Howard and Brooks-Gunn, suggesting that supportive relationships can counteract some of the negative impacts on parenting.^121^

### Maternal childhood trauma and the influence on parenting practices

Among the participants experiencing the different syndemics, our study identified that traumatic experiences from childhood or adolescence were commonly reported across all the groups. These early traumas appear to contribute to challenges into adulthood. Research substantiates this, showing that childhood trauma can lead to mental health conditions such as depression, anxiety and PTSD into adulthood.^122^,^124^ These mental health challenges, in turn, can exacerbate vulnerabilities to IPV and HIV, forming a complex web of interconnected challenges. Additionally, trauma has been linked to risky sexual behaviours, further increasing the risk for both HIV transmission and additional instances of trauma, such as IPV or sexual assault.^125^

Quantitative research has already highlighted the significant impact of childhood trauma on parenting behaviour, further emphasising the need for tailored interventions for caregivers who are survivors of such experiences.^126^ In line with existing literature on the consequences of childhood sexual abuse on maternal parenting,^127^ our study revealed a strong tendency for overprotective parenting among mothers from the IPV-Mental Health-HIV and HIV-Mental Health groups. This corroborates numerous studies that link childhood sexual abuse to overprotective behaviours as mothers often feared that their children would experience what they had experienced in childhood.^128 129^ Research among women with experiences of childhood sexual abuse found that these mothers were more anxious, often experiencing post-traumatic stress that manifested as hypervigilance.^128^ The state of hypervigilance can lead to overprotective behaviours where mothers became excessively cautious and protective of their children.^128^ Parenting tasks and the development stages of the children can trigger memories and emotions related to their own abuse.^129^ For instance, when a child reaches the age at which the mother was abused, this can be triggering, leading to increased protectiveness, this fear can lead to behaviours such as constant monitoring, restricting the child’s interactions with others and being overly cautious about physical touch and affection.^129^

However, the tendency towards overprotectiveness was less pronounced among mothers in the IPV-Mental Health group. This discrepancy could be attributed to a number of factors, including the strained relationships with their children’s fathers and the ongoing abusive relationships that most were still in. Notably, unlike mothers in the HIV-IPV-Mental Health group, most mothers in the IPV-Mental Health cohort were living with their children. This living arrangement could also have contributed to their reduced protective instincts, perhaps because the constant proximity diminished the perception of an immediate threat to their children, unlike the HIV-IPV-Mental Health group where physical separation might have heightened concerns for their children’s well-being.

Additionally, our study revealed that mothers and caregivers generally did not report bonding challenges with their children, except in the IPV-Mental Health group, where participants reported these challenges. This finding is consistent with previous research which found that women that were traumatised by experiences of IPV reported more difficulties bonding with their children.^119^

### Mothers’ and caregivers’ socioeconomic challenges and parenting outcomes

Our study reveals that women living with the syndemic experienced socioeconomic challenges, which also had an influence on their parenting practices. The interaction between epidemics and poverty forms a self-reinforcing cycle.^11 130 131^ Specifically, mental health conditions like depression and anxiety can diminish employability and job retention, affecting productivity and motivation.^132^ IPV further exacerbates these issues, as controlling behaviours from an abusive partner and subsequent mental health symptoms can undermine employment stability.^133^ Likewise, an HIV-positive status introduces additional employment barriers such as employer stigma and inflexible work schedules that can prevent employers from going to the clinic.^134^ Cumulatively, these factors heighten the risk of unemployment and lower socioeconomic status.

In terms of parenting, our findings align with existing studies which have shown that economic hardship amplifies stress and inter-parental conflict.^135^ In our study this was particularly evident among the IPV-Mental Health group. Additionally, parents caught in the syndemic (HIV-Mental Health-IPV, IPV-Mental Health, HIV-Mental Health) often fail to meet their children’s essential needs, compromising their caregiving role and intensifying poverty-induced stress.^136^ This economic strain fosters increased parental stress, heightening the risk of poor mental health and, consequently, the adoption of harsher parenting practices.^135 137^ A review of the relationship between poverty and child abuse and neglect found that poverty is a significant risk factor for child maltreatment.^138^ Both familial and community-level poverty, as well as economic insecurity, have been strongly associated with an increased risk of child abuse and neglect.^138^ Economic hardships can lead to psychological distress, relationship problems and disrupted parenting, which in turn can increase the likelihood of child abuse.^138^

### Limitations

It must be noted that the current study had some limitations. While participants were asked in general about violence they may have experienced in their childhood, we did not specifically ask about child sexual abuse. This may have led to some participants in the other groups (HIV-Mental Health and IPV-Mental Health) choosing not to disclose such experiences. Moreover, the sensitive nature of the topics that were discussed as well as the stigma attached to people living with the syndemic, could have influenced participants’ willingness to fully share and be open about certain topics such as the use of corporal punishment especially among the IPV-Mental Health-HIV group. This may have led to potential under-reporting. However, to address this, interviewers assured the participants that all information would be kept confidential, and they were offered psychosocial support from the social worker if they felt distressed during the interview. Finally, the findings might be most relevant to the specific cultural and geographic settings of the study, possibly limiting their broader relevance. However, this research remains essential for comprehending the lived realities of women in certain South African provinces and can pave the way for more extensive, varied research.

### Implications for practice and future research

The findings of our study have important implications for designing parenting interventions, particularly for mothers living with various syndemic conditions. When developing interventions, it is essential to consider the specific epidemics women are living with, as well as various associated factors such as socioeconomic status, parental relationships and maternal experiences of childhood trauma.

Addressing socioeconomic challenges is critical. Although South Africa offers social welfare grants to economically disadvantaged families, our research reveals that these grants were not always paid out regularly. Additionally, the child support grant was often inadequate to meet the family’s needs. We recommend increasing the amount of the child support grant to better support families, particularly to enable unemployed mothers to provide for their children’s basic needs and overall well-being.

Furthermore, parenting interventions need to harness the social support from family members by involving grandparents and aunts in sessions.^57^ Additionally, it is crucial to address strained relationships between parents recognising the three-way relationship of parenting. Even in cases where there is no IPV, relationship strain and conflict can have a severe impact on parenting.

Moreover, research has recently started to recognise the intersection of VAC and violence against women.^139^ Although interventions have tended to focus predominantly on gender relations and ending IPV, they often neglect the impact of poor mental health on the victims as well as the perpetrators. Given the prevalence of poor mental health in these contexts, and their persistence even after IPV has ceased, it is crucial to intentionally integrate a mental health focus into interventions. The persistence of mental health challenges even after the dissolution of a violent relationship can lead to continued violence against the child.^140 141^ A good example of integrated interventions are those developed by Equimundo, which include a focus on addressing the psychological impact of IPV on children and women, and harmful gender norms and social learning in an attempt to curb the intergenerational transmission of violence.^142^

Finally, our research points to a noticeable gap in understanding the intricacies of motherhood within the context of these overlapping epidemics. Particularly, there’s a lack of knowledge concerning mothers who are not living with their children due to the syndemic and this is a common phenomenon in South Africa. This warrants further mixed-methods research to explore the unique challenges and circumstances these women face and the impact on the parent–child relationship.

These recommendations serve as practical implications that can guide both interventions and future research directions, aimed at mitigating the complex socioeconomic and parenting issues exacerbated by the syndemic.

## Conclusion

In conclusion, our study highlights that living with the different combinations of the HIV-IPV-Mental Health syndemic complicates parenting. Mothers facing these overlapping challenges often lose custody, struggle with relational issues with their children’s fathers, and engage in less effective parenting practices. Additionally, the syndemic intensifies socioeconomic hardships, further eroding parenting quality by causing stress and unemployment. In essence, the syndemic not only makes parenting more difficult but also perpetuates a cycle of disadvantage for both parents and children. Developing interventions that take into consideration the associated factors of living with these epidemics and how they impact parenting is critical.

## Data Availability

Data are available upon reasonable request.
