# Supplementary material for: Qualitative study exploring parenting among mothers and female caregivers living with the IPV, mental health and HIV syndemic in South Africa
Source: BMJ Open. 2024 Oct 29;14(10):e086478. doi: 10.1136/bmjopen-2024-086478 (PMC11529702; doi:10.1136/bmjopen-2024-086478)
Supplement: Supplementary file 1 [file bmjopen-2024-086478.reviewer_comments.pdf]

## PEER REVIEW HISTORY

BMJ Open publishes all reviews undertaken for accepted manuscripts. Reviewers are asked to complete a checklist review form and are provided with free text boxes to elaborate on their assessment. These free text comments are reproduced below.

## ARTICLE DETAILS

| | |
|---|---|
| **TITLE (PROVISIONAL)** | A qualitative study exploring parenting among mothers and female caregivers living with the IPV, mental health and HIV syndemic in South Africa |
| **AUTHORS** | Silima, Mpho; Christofides, Nicola; Franchino-Olsen, Hannabeth; Woollett, Nataly; Meinck, Franziska |

## VERSION 1 - REVIEW

| | |
|---|---|
| **REVIEWER NAME** | ***Mangum, Laurenia*** |
| **REVIEWER AFFILIATION** | University of Illinois Chicago |
| **REVIEWER CONFLICT OF INTEREST** | Na |
| **DATE REVIEW RETURNED** | 04-Jun-2024 |

| | |
|---|---|
| **GENERAL COMMENTS** | Overall, this was a well-written and impactful paper on such an important topic that will provide the field with a greater understanding of the lived experiences of mothers who experience IPV, poor mental health, and/or are living with HIV. We see the act of parenting emerge not just a gendered role but a huge undertaking in the context of childhood trauma, sexual violence, and gender inequalities. I thank the authors for conducting such important work and am equally appreciative for the opportunity to review this manuscript.<br><br>Here I offer ways to improve the readability of the manuscript and ways to enhance the methodological rigor of the paper.<br>Introduction and Background<br>1. As it stands, the paper is long and the beginning of it loses the audience before we get to the methodology.<br>2. Consider removing the paragraph "parenting is a complex and multifaceted concept" .. and begin with "In South African, women . . ."<br><br>Methodology<br>3. Study design- There is no mention of an inductive vs deductive qualitative approach- although it appears to be deductive since the authors are using syndemics theory.<br>Study setting- is there new epidemiological data that can be cited (population, HIV prevalence, unemployment rates)? The citations here are from 2016, 2017.<br>4. No mention of confirmability, transferability, authenticity, credibility. Positionality is missing for each member of the study team.<br><br>5. Inclusion and exclusion criteria is missing?<br>6. How many questions were in the interview guide?<br>7. Who interviewed the women?<br>8. The COREQ should be updated to provide this detailed |

| | |
|---|---|
| | information and not refer the reader to the page as page numbers change and the information is outlined in detail. e.g. Was anyone else present besides the participants and researchers? You could put the children and other family members.<br>9. Compensation for participation?- should be moved to data collection section.<br>10. If data saturation was not discussed among the study team, how do you know that you have reached penetration of the themes?<br><br>11. How were the interviews captured? Recorder? How were they transcribed and analyzed?- if this information is later stated, it should be moved to the methods section in data collection and data analysis.<br>12. The interviews were conducted in the local language. Describe the process of translating the transcripts from the local language to English and the processes undertaken to maintain the meaning of cultural phrases and nonverbal communication. - This was placed in the limitations but should be discussed in the methods section.<br>13. include the number of items of each scale that was used "Ask Suicide Screening Questions (ASQ) items?", GAD, items, PHQ-9? PTSD-8 is it 8 items?<br><br>14. Who trained the facilitators of the arts-based methods? Why was only one person the decider if the method should be administered more than once or not? Make the justification<br>15. Who are the members of the research team?<br>16. Revisit ethical considerations to move some information to other sections (compensation).<br>A grocery card is not a token of appreciation, it is compensation for participating in a research study. Why was zar50 an adequate payment?<br>17. Coding and analyzing data are two distinct steps. Clearly outline the coding process and then talk about analyzing the data.<br>18. Reflexivity is minimally provided. Expand this section<br>19. The results section uses different formatting from the rest of the paper. Make that section "Results" and then go into the sample sociodemographic and characteristics<br>20. What is the mean age?<br>21. Do you have information on how long they’ve been living with HIV?<br>22. Give us a brief summary of the themes generated prior to discussing them. The four themes generated included x,y,z,a.<br><br>23.The authors offer a little review of parenting but do not provide an understanding of parenting from the cultural perspective of South Africa. Consider revising this section of the background to add this. What is the cultural significance. It will help when the readers get to the results to contextualize the findings.<br><br>24.Which group is Halo part of? It’s unclear here. Page 15<br><br>25. Expand the lit review to discuss childhood trauma and the prevalence of sexual abuse<br><br>26. I would encourage the authors to rethink the naming of the themes generated. Theme 1 living vs not living doesn’t quite capture the essence of the process mothers are enduring for the betterment of their children. They're sacrificing the autonomy of raising their children due to financial strains. They are also embattled by gender inequalities- sexual violence. |

| | |
|---|---|
| | 27. Provide an expanded discussion of cultural differences in child rearing and corporal punishment for Black mothers and caregivers. Provide a greater contextual foundation.<br><br>28. Childhood trauma- this theme doesn't adequately capture the essence of the quotes. Again, gender inequality, parentification of girl children<br><br>29. What is the grant that the women speak of? Provide the reader of an understanding of the welfare benefits.<br><br>30. Consider reviewing the quotes for readability; there are a few that should be edited for English grammar. Very minor but noticeable.<br><br>Discussion- page 21<br>31. Underlying theme is the gender-based violence impacting these women. Over protection is a manifestation of post-traumatic stress, hyper vigilance. This is vaguely discussed.<br><br>32. The study discusses attachment theory, however, this theory wasn't discussed in the literature review- consider removing or address early on in the literature review. Same with family stress theory.<br><br>33. It may be a good idea to map out the conceptual framework to clearly delineate how variables are impacted each other. Poverty is a structural determinant whereas HIV, IV, and MH are psychosocial factors. Poverty can be exacerbated and underlying but not appear as a result of experiencing these things. upward mobility can be impacted. Yes, household income, yes. But not poverty<br><br>34. Be careful of using causal language to describe qualitative data Bottom #26. I don't think you can say poverty causes physical abuse but rather multistressed parents may invoke corporal punishment more than those without those stressors<br>35. The question about disciplining the children wasn't asked in the context of stress and resolving conflict in the home thus the inference made is out of the context of the research question and aims.<br>36. Address the translation in the methods section<br>37. The study is also about supportive family members but does not delve into social support. Revise that as it was not the main focus of this study. |

| | |
|---|---|
| **REVIEWER NAME** | ***Eaton, Andrew*** |
| **REVIEWER AFFILIATION** | University of Regina, Faculty of Social Work - Saskatoon Campus |
| **REVIEWER CONFLICT OF INTEREST** | Na |
| **DATE REVIEW RETURNED** | 21-Jun-2024 |

| | |
|---|---|
| **GENERAL COMMENTS** | Article summary<br>- Limitations point 2 could be more specific as "arbitrary decisions" is unclear. Perhaps something like "Although validated scales for MH were used to identify participants with MH this may have introduced some arbitrary decisions being made." |

| | |
|---|---|
| | Methods & Results<br>- In parts, this is referred to as a narrative study. In others, as an arts-based one. Please make consistent.<br>- Thematic analysis, particularly as described in the ms, seems insufficient for identifying underlying mechanisms for syndemics from arts-based, narrative interviews. The risk of overinterpretation seems very present.<br>- Further to this point, I don't see mechanisms identified in the Results. I see stories, which fits the methodological approach of narrative arts-based work. I recommend removing reference to mechanisms, as the methods and results don't support this claim.<br><br>Overall<br>- There are numerous acronyms that could confuse a reader. I recommend spelling out "mental health" and "South Africa" each time, and encourage the authors to further trim the amount of acronyms.<br>- The manuscript would benefit from careful proofreading as there are repeated words (e.g., "syndemic syndemics" in the Abstract), "data" treated as a singular term, and other issues (e.g., repeating "in-depth" twice in a single sentence).<br>- The in-text citations and references don't appear to be in Journal style.<br>- Some claims are missing citations (e.g., page 7, lines 12-14). |

## VERSION 1 – AUTHOR RESPONSE

**Reviewer 1**
**Overall, this was a well-written and impactful paper on such an important topic that will provide the field with a greater understanding of the lived experiences of mothers who experience IPV, poor mental health, and/or are living with HIV. We see the act of parenting emerge not just a gendered role but a huge undertaking in the context of childhood trauma, sexual violence, and gender inequalities. I thank the authors for conducting such important work and am equally appreciative for the opportunity to review this manuscript.**
**Response**
**Thank you, Reviewer 1, for your complimentary response on our work and your constructive feedback which has made this a much stronger paper.**
**1. As it stands, the paper is long and the beginning of it loses the audience before we get to the methodology. 2. Consider removing the paragraph "parenting is a complex and multifaceted concept" ... and begin with "In South African, women.**

**Thank you for this suggestion, the introduction now reads:**
**Introduction**
**In South Africa, women disproportionately bear the burden of HIV, intimate partner violence (IPV), and poor mental health, which can significantly affect their parenting.1–3 Syndemic theory offers a lens to understand how these overlapping epidemics interact and amplify health burdens.4 This theory posits that multiple comorbidities, fuelled by structural and environmental factors, have a synergistic effect that worsens the overall health impact on a population more than the sum of individual conditions.4 Singer's early work on syndemic theory primarily focused on the co-occurrence of substance abuse, violence, and AIDS (SAVA) among marginalized communities in the United States. 4 The current study expands on SAVA**

to include poor mental health. This expansion is not new, as Tsuyuki and colleagues (2017) studied the syndemic of substance use disorder, violence, and mental health among HIV-infected individuals.5 However, very little research has investigated the impact of these syndemics on parenting practices.

## Conceptual Framework: IPV, Mental Health and HIV as a syndemic

Existing literature confirms the frequent co-occurrence of the IPV, poor mental health and HIV epidemics.6–9 The relationship between HIV and poor mental health is bidirectional, meaning that HIV can lead to poor mental health outcomes, and poor mental health can increase the risk of acquiring HIV. 10,11 The high prevalence of poor mental health conditions among people living with HIV is often associated with poorer physical health, lower quality of health services, and poor adherence to antiretroviral treatment (ART). 12–14 Furthermore, having poor mental health can increase the risk of HIV acquisition through both direct and indirect mechanisms.15 Adolescents and adults with serious mental illnesses who are sexually active may be more likely to engage in high-risk behaviours, including inconsistent condom use, having multiple sexual partners, engaging in transactional sex and consuming alcohol before sex, which may increase the likelihood of unprotected sex.16–18 Additionally, IPV can precipitate mental health challenges such as PTSD, anxiety, depression, suicidal thoughts, and substance use disorder.19–23 Studies have shown that women experiencing IPV are at higher risk of developing these mental health challenges due to the chronic stress and trauma associated with ongoing abuse, as well as the possible isolation from social support systems.24–26 Conversely, poor mental health can make individuals more susceptible to IPV due to factors such as low self-esteem, emotional dependency, and difficulty recognizing abusive behaviours. 27,28Moreover, the relationship between IPV and HIV involves both biological and socio-cultural pathways.29 Biologically, forced or coercive sexual intercourse with an HIV-infected partner can lead to HIV and other sexually transmitted infections.30 Socio-culturally, violence and the fear of violence compromises the women’s ability to negotiate condom use, which can lead to inconsistent condom use and thereby increase HIV risk. 31

An important aspect of syndemic theory is that structural and environmental factors impact the disease clustering and often exacerbate the syndemic conditions. 4,32 These include poverty, gender inequality, stigma, and lack of access to resources and services that could improve the health and wellbeing of individuals. 33,34 Financial stress and lack of resources can exacerbate mental health challenges, due to the higher levels of stress as a result of financial insecurity, job instability, and poor living conditions, which are significant risk factors for depression and anxiety.35 Furthermore, financial dependence on a partner can significantly reduce one’s bargaining power within the household making it difficult to leave an abusive situation.36 In addition, people living in poverty may engage in risky sexual behaviours to meet their basic needs, which can elevate their risk of contracting HIV.37,38 Cultural factors, such as gender norms and expectations, also influence the epidemics.39–41For instance in many societies, traditional gender roles can reinforce power imbalances, making women more vulnerable to IPV and less able to negotiate safe sex practices, increasing their risk of HIV.41 Additionally, stigma associated with IPV, poor mental health, and HIV can hinder individuals' willingness to seek help and adhere to treatment leading to further isolation, untreated conditions, and exacerbation of both physical and mental health challenges.42–44

Other factors, such as adverse childhood experiences, can also increase the risk of the co-occurrence of the epidemics. Additionally, adverse maternal experiences in childhood not only elevate the risk of poor mental health in adulthood but also increase the likelihood of experiencing IPV and acquiring HIV 26,45, which, as previously noted, significantly impact parenting practices. Child sexual abuse is alarmingly prevalent in South Africa.46 A nationally

representative cross-sectional study among 5631 households found that 9.99% of boys and 14.61% of girls reported experiencing sexual violence in their lifetime, with physical abuse, emotional abuse, and neglect strongly associated with sexual violence.46 Trauma from child abuse is a significant factor contributing to adult mental health challenges such as PTSD, depression, and anxiety.26,45,46The mental health challenges as a result of the abuse may increase the survivors' risk of being involved in violent relationships and as a result elevate their HIV risk. 26,47 Survivors of abuse often struggle with establishing healthy relationships due to the trauma, which can lead to low self-esteem, emotional dependency, and difficulty in setting boundaries. 26 This dynamic, combined with the impaired ability to negotiate safe sex, significantly heightens the risk of HIV infection among these individuals.47

Parenting practices among women living with the HIV, IPV, and Poor Mental Health syndemic
Parenting is influenced by various factors, including the characteristics and experiences of parents and the context in which they are raising their children.48 Mothers living with HIV encounter unique challenges.49,50 Some evidence indicates a heightened risk of psychiatric vulnerability among women with HIV, especially in sub-Saharan Africa,51 which is exacerbated by disease-related stress and structural challenges such as poverty, 52,53 gender inequity,51 and limited mental health services.54 Maternal HIV can negatively impact both infected and uninfected children's developmental, psychological, and physiological well-being.55 These adverse outcomes arise partly from compromised parenting due to maternal stress and mental health deterioration, potentially leading to neglect or aggression towards the child.56 This is especially significant in sub-Saharan Africa, where 1.36 million pregnant women were living with HIV as of 2010.57
IPV further complicates the parenting landscape. High rates of IPV among heterosexual couples indicate that many children are raised in violent households.58 Early exposure to IPV negatively impacts children's mental health, resulting in trauma, depression, and anxiety.59,60 A meta-analysis of 118 studies revealed a 50-63% likelihood of children developing internalizing problems and clinical disorders due to violence exposure.61 IPV impacts children's emotional regulation and introduces environmental stressors that may result in mood swings and low self-esteem among the children, thereby making the parenting experience more difficult.62,63 Additionally, mothers experiencing IPV are more likely to resort to harsh discipline, sometimes escalating to physical abuse, both to placate their abusers and as a result of displaced aggression. 64 The emotional toll, often manifesting as depression, affects the quality of mother-child interactions and can erode mothers' confidence in their parenting capabilities. 65,66
Poor mental health adds another layer of complexity to parenting. Parental depression is significantly associated with more hostile and harsh parenting.67,68 With 10-20% of women experiencing depression in their lifetime and more than 30% having persistent depressive symptoms.69,70
It must be noted, although HIV, IPV, and poor mental health can lead to harsh parenting, there are protective factors that can buffer these socio-emotional outcomes. Resilience, social protection, and the ability of some mothers to act as emotional anchors during moments of violence or distress can mitigate the impact of IPV. 71,72 For mothers living with HIV and poor mental health, access to antiretroviral therapy, social support, and effective mental health services can improve parenting quality and mitigate negative impacts on children.73,74

The cultural significance of parenting in South Africa
Parenting practices in South Africa are deeply influenced by cultural norms and values.75 The extended family often plays a crucial role in parenting, with grandparents, aunts, uncles, and older siblings actively involved in the upbringing of children. 75–77 According to Statistics South Africa (2023), households with more than four children are more likely to have children cared for by extended family members (84.5%). Furthermore, the same survey found that a fifth

**of all children did not live with both their parents, and most children lived with only their mothers and other extended family members (42.0%).77 This collective approach to parenting is rooted in the African concept of "Ubuntu," which emphasizes compassion for others and the interconnectedness of all people.78 Traditional gender roles also influence parenting, with women generally seen as the primary caregivers responsible for day-to-day nurturing and education of children, while men provide financial support.76 Fragmented families in South Africa can be attributed to labour migration and low marriage rates especially among African women, who are also less likely to live with the child's father if they are not married.76,79 The historical and socio-political context of South Africa, particularly the legacy of apartheid and ongoing socio-economic challenges, significantly shapes parenting practices.80 The psychological impact of the apartheid-era trauma has affected parenting as parents who experienced trauma may struggle with emotional regulation, influencing their ability to engage in consistent and nurturing practices.80 Additionally, during colonialism, corporal punishment was institutionalized as a method of maintaining order and discipline in both schools and homes.81 As a result, many South African homes today still view corporal punishment as integral to teaching children respect for authority and obedience.81,82 Despite the legislative ban on corporal punishment, many mothers continue to practice this form of parenting.83 A qualitative study found that parents disagreed with the law and held positive attitudes towards corporal punishment, viewing it as effective for disciplining children.82 Mothers often used corporal punishment to correct children's behaviour, lacking knowledge of alternative disciplinary methods.82 The use of corporal punishment is deeply embedded in cultural and historical contexts, passed down through generations as a normative practice.82**

**Present Study**
**The current study aims to explore parenting among women living with the different syndemics (specifically, HIV-Mental Health; IPV-Mental Health; and HIV-Mental Health-IPV) and how these syndemic conditions influence parenting practices. This research moves beyond examining each syndemic in isolation to highlight how their interrelated and compounded effects further complicate parenting. In a context where millions of children and mothers grapple with these complex epidemics, a nuanced understanding of these interactions offers valuable points for targeted interventions.**

**2. Study design- There is no mention of an inductive vs deductive qualitative approach- although it appears to be deductive since the authors are using syndemics theory.**
**Under the data management and analysis section we have now included information on the deductive approach that was used, it now reads:**
**A typology of participants was developed deductively using the syndemic theory. A broad coding framework was developed based on the research objectives and an initial thematic review of selected transcripts.104This framework was informed by key concepts from syndemic theory, such as the interactions between IPV, mental health and HIV and the contextual factors that lead to the clustering of the epidemics, and served as a guide for coding the data.34 We then identified inductive codes based on a deep reading of the transcripts and by comparing and contrasting the findings between the categories of participants based on the syndemic theory.**

**Thematic analysis, following the six-step process outlined by Braun and Clarke (2006), was used to analyse the interviews.105 The steps included familiarizing ourselves with the data, searching for emerging themes by examining the coded data to identify broader patterns of meaning, reviewing themes to ensure that they accurately reflected the data, and applying the themes to the data during the write-up.106**

**3. Study setting- is there new epidemiological data that can be cited (population, HIV prevalence, unemployment rates)? The citations here are from 2016, 2017.**
**Thank you for highlighting this point, Reviewer 1. We have now updated the study setting section with the most recent epidemiological data available for HIV and IPV. Unfortunately, there is no recent data on poor mental health in Mpumalanga. The updated section now reads as follows (In manuscript, in cite referencing is in Vancouver):**
**We conducted the study in two sites within Mpumalanga Province, South Africa, from October 2022 to February 2023. In 2022, Mpumalanga Province reported an estimated population of approximately 4,720,497 people (Statistics South Africa, 2022). The primary languages spoken include siSwati (27.67%), isiZulu (24.1%), Xitsonga (10.4%), and isiNdebele (10%) (South African High Commission (Ottawa), 2019). In 2019 there were 1.26 million people living in poverty across the district (Department of Cooperative Governance & Traditional Affairs, 2020), with high levels of unemployment at 36.69% (Department of Cooperative Governance & Traditional Affairs, 2020). Recent data based on a population-based household survey indicated an HIV prevalence of 20.8% among adults aged 15 years and above (Human Sciences Research Council, 2023), with approximately 38% physical IPV prevalence among girls and young women in Mpumalanga (Leddy et al., 2019).**
**4. No mention of confirmability, transferability, authenticity, credibility. Positionality is missing for each member of the study team.**
**Response:**
**Thank you, reviewer 1 for this suggestion, we have added a section in the methodology titled Research Trustworthiness that provides a detailed discussion on confirmability, transferability, authenticity, credibility and positionality**
**To ensure the quality of the research we adhered to the criteria of credibility, transferability, dependability and confirmability. 103 Credibility was ensured through deep engagement with the local community. Our qualitative interviewers spent five months in the community, engaging with the participants to build trust and gather rich data. Prior to the qualitative interviews, the main study and quantitative interviewers had also established rapport with the participants, having been in the community for two years. The research team was highly qualified and interdisciplinary, including a social worker, a trauma therapist, and experts on violence against children, IPV research, and community engagement. To support peer debriefing, the management team held weekly sessions with the interviewers. These sessions allowed the team to discuss ongoing interviews, strategize, and address any challenges related to data collection and provide emotional support to the interviewers. To ensure dependability, a detailed proposal was developed outlining the qualitative data collection process, and the interviewers maintained thorough field notes. Intercoder agreement and reliability were ensured through team reviews of the initial six transcripts, guaranteeing consistent application of the codes.**
**To ensure confirmability, reflexivity played a critical role. Weekly debriefing meetings served as a platform for the research team to reflect on the research process, discuss any biases, and examine how our backgrounds might influence data collection and interpretation. Field notes were taken following each interview to assist in the research process, serving as a record of the researcher's thoughts and reactions. These notes facilitated ongoing reflection on how the researcher's role might influence the study's findings and interpretations. Furthermore, each team member continually reflected on their positionality. The research team comprised of individuals with diverse backgrounds, including two black women with post graduate qualifications (one Master's student and a PhD candidate), one black man who was a Masters student, and three white women with PhD qualifications. The researchers acknowledged their middle-class status and post-graduate qualifications while working in these communities marked by severe poverty. This self-awareness was essential to understand how our socio-economic status, race, gender and educational background could impact our interactions with participants and the data collected. For instance, being interviewed by a man may have**

influenced the willingness of some of the female participants to disclose certain experiences. By actively reflecting on these factors during the weekly debriefing meetings and throughout the research process, the team aimed to mitigate potential biases and enhance confirmability of the study's findings. To ensure transferability, we provided detailed descriptions of the study context and participant characteristics, allowing readers to understand whether the findings might apply to their own settings.

5. Inclusion and exclusion criteria are missing.
The inclusion and exclusion criteria are in the section that describes the sample. We have now edited this section to explicitly describe what the exclusion and inclusion criteria was, it now reads:
Sample
A purposive sampling approach was used to recruit twenty women between the ages of 20 and 60 years, using specific survey items from the Interrupt_Violence Study as the screening criteria.91 These survey items assessed participants' experiences with HIV, poor mental health , and IPV. The inclusion criteria required the women to have experiences with at least two of the three epidemics: IPV, poor mental health or HIV. The experiences of IPV could be current or past, while poor mental health needed to be current. Poor mental health was assessed using several tools: the four-item Ask Suicide Screening Questions (ASQ) 92, the seven-item Generalized Anxiety Disorder Questionnaire (GAD-7) 93; the nine-item Patient Health Questionnaire (PHQ-9) for depression 94 and the 8-item Post Traumatic Stress Disorder Questionnaire (PTSD-8).95 For IPV, participants had to respond "yes" to any of the questions in the World Health Organization instrument 96 for physical, emotional and sexual violence victimization experiences. HIV status was either self-reported by the participant or assessed through voluntary HIV testing conducted by the fieldworker. Additionally, participants had to self-identify as caregivers for a child or children under the age of 17. This was determined by asking participants in the survey, "For how many children are you a caregiver for like a parent?" Participants who answered more than zero were further asked about their relationship with the child, with common responses being mother, aunt or grandmother. Men were not included in the study because it is usually women who are the primary caregivers for children in South Africa and who are disproportionately affected by the epidemics under investigation

6. How many questions were in the interview guide?
The interview guide had twenty questions, with multiple probes. We have added this in the section under data collection tools and procedure and it now reads:
Through in-depth open-ended interviews using an interview guide comprising of twenty questions, we were able to elicit in-depth narratives from the participants, capturing their perspectives, emotions around parenting.
7. Who interviewed the women?
We have added the following sentence in the data collection section to reflect who interviewed the women.
All the interviews were conducted face-to-face by two research assistants and a PhD candidate, either in the participants' homes or at a location of their choice, with each interview lasting 60 to 90 minutes.
8. The COREQ should be updated to provide this detailed information and not refer the reader to the page as page numbers change and the information is outlined in detail. e.g. Was anyone else present besides the participants and researchers? You could put the children and other family members.
Thank you for this suggestion, we updated the COREQ to include detailed information directly within the checklist. This will ensure clarity and avoid referencing specific page numbers, which may change. However, please note that in some instances, there is limited space to

provide more detail. We have included as much relevant information as possible within the given constraints. Thank you for your recommendation.
9. Compensation for participation? should be moved to data collection section.
Thank you for noting this, we have moved compensation for participation under the data collection section, and it now reads:
Participants were given a ZAR50 ($2.71) grocery store gift card as compensation for their participation in the study.
10. If data saturation was not discussed among the study team, how do you know that you have reached penetration of the themes?
Thank you for this comment Reviewer 1, we have added the following to the manuscript as well.
Data saturation was assessed through the iterative process of data collection and analysis. This iterative process involved collecting and analysing the data in cycles. After each round of data collection, the data was immediately reviewed and analysed to identify emerging themes and patterns. Based on this preliminary analysis, subsequent data collection efforts were adjusted to explore the themes in greater depth or to investigate any new areas that may arise. After each round of interviews, the research team reviewed the findings to identify recurring themes and patterns. As data collection progressed, we observed that no new themes or insights were emerging, and the information provided by participants began to repeat. This repetition, or redundancy, indicated that we were not gaining any new insights from additional data, suggesting that data saturation had been reached.
Moreover, the consistency of the themes across multiple interviews reinforced our confidence that we had comprehensively captured the participants' experiences. While formal discussions on data saturation were not explicitly conducted among the study team, the iterative nature of our data collection and analysis provided a clear, practical indication that data saturation had been achieved.
11. How were the interviews captured? Recorder? How were they transcribed and analyzed?- if this information is later stated, it should be moved to the methods section in data collection and data analysis.
Information on the audio recording, translation and transcription of the interviews was included in the data analysis section, it has now been moved to the data collection section and reads as below:
Interviews were audio recorded, translated and transcribed verbatim by trained transcribers.
12. The interviews were conducted in the local language. Describe the process of translating the transcripts from the local language to English and the processes undertaken to maintain the meaning of cultural phrases and nonverbal communication. - This was placed in the limitations but should be discussed in the methods section.
A discussion on translation and transcription processes has been added in the methods section and it now reads:
The local languages of Xitsonga and SiSwati were used during the interviews which were audio recorded, translated, and transcribed verbatim by trained transcribers. The translation process was rigorous to ensure the accuracy and cultural relevance of the transcripts with bilingual transcribers, fluent in both the local languages and English who were also responsible for the translations. In instances where there was no equivalent word in English, the transcribers included the siSwati or Xitsonga word and provided an explanation in English to maintain the meaning of cultural phrases. Additionally, the research team reviewed the translated transcripts alongside the original audio recordings to verify the accuracy of the translations
13. include the number of items of each scale that was used "Ask Suicide Screening Questions (ASQ) items?", GAD, items, PHQ-9? PTSD-8 is it 8 items?
The number of items for each scale has been added, this now reads:
Poor mental health was assessed using several tools: the four-item Ask Suicide Screening

Questions (ASQ) 92, the seven-item Generalized Anxiety Disorder Questionnaire (GAD-7) 93; the nine-item Patient Health Questionnaire (PHQ-9) for depression 94 and the 8-item Post Traumatic Stress Disorder Questionnaire (PTSD-8).95

14. Who trained the facilitators of the arts-based methods? Why was only one person the decider if the method should be administered more than once or not? Make the justification

One of the co-investigators trained the interviewers on the arts-based methods, this has been added in the manuscript for better clarity for the reader. Furthermore, the justification for using more than one method with a participant was decided during the interview and was decided on a case by case basis by the specific interviewer, this was only decided in a situation where the interviewer felt that there was more information that could be gathered by using a different method, or if the participant was struggling with the first method.

This section has been edited for better clarity and it now reads:

The interviewers were trained in the arts-based methods by one of the co-investigators, a therapist and researcher with expertise in psychology, play therapy, and art therapy.

The decision to have some participants engage in more than one activity was made on a case-by-case basis by the interviewer during the interview. This decision was based on two main considerations, firstly if the participant was struggling with an activity, a less challenging one was introduced to facilitate their engagement, and if the interviewer felt that there would be additional valuable information that could be gathered through another method. The interviewer, being directly engaged with the participant was best positioned to make these decisions in real-time to ensure the effectiveness and comfort of the participant. This approach allowed for flexibility and responsiveness to each participant's unique needs and circumstances, ensuring richer and more comprehensive data.

15. Who are the members of the research team?

The research team included the research assistants and study investigators. This has been clarified in the section under data management and analysis and now reads:

A codebook was created and shared with the research team, A codebook was created and shared among the interviewers and study investigators for discussion.

16. Revisit ethical considerations to move some information to other sections (compensation).

Some of the information in the ethical considerations has been moved, this includes information on transcription of the audio recording as well as the compensation this has all been moved to the data collection section.

A grocery card is not a token of appreciation, it is compensation for participating in a research study. Why was zar50 an adequate payment?

Thank you, reviewer, 1, you are correct this is a compensation for their participation, the wording has been edited to reflect this and we have also added the justification for why ZAR50 was considered adequate payment, the section now reads:

Participants were given a ZAR50 ($2.71) grocery store gift card as compensation for their participation in the study. This amount was chosen as it is not deemed to be coercive in the setting (rural and peri-urban Mpumalanga).

17. Coding and analyzing data are two distinct steps. Clearly outline the coding process and then talk about analyzing the data.

Thank you, reviewer, 1, this section has been edited to reflect the two distinct steps of coding and then analysis. It now reads:

A typology of participants was developed deductively using the syndemic theory. A broad coding framework was developed based on the research objectives and an initial thematic review of selected transcripts.104This framework was informed by key concepts from syndemic theory, such as the interactions between IPV, mental health and HIV and the contextual factors that lead to the clustering of the epidemics, and served as a guide for coding the data.34 We then identified inductive codes based on a deep reading of the transcripts and by comparing and contrasting the findings between the categories of

participants based on the syndemic theory.

Thematic analysis, following the six-step process outlined by Braun and Clarke (2006), was used to analyse the interviews.105 The steps included familiarizing ourselves with the data, searching for emerging themes by examining the coded data to identify broader patterns of meaning, reviewing themes to ensure that they accurately reflected the data, and applying the themes to the data during the write-up.106

18. Reflexivity is minimally provided. Expand this section

More information on reflexivity has been incorporated into the sub-section "Study Trustworthiness" this now reads:

To ensure confirmability, reflexivity played a critical role. Weekly debriefing meetings served as a platform for the research team to reflect on the research process, discuss any biases, and examine how our backgrounds might influence data collection and interpretation. Field notes were taken following each interview to assist in the research process, serving as a record of the researcher's thoughts and reactions. These notes facilitated ongoing reflection on how the researcher's role might influence the study's findings and interpretations. Furthermore, each team member continually reflected on their positionality. The research team comprised of individuals with diverse backgrounds, including two black women with post graduate qualifications (one Master's student and a PhD candidate) one black male Masters student and three white women with PhD qualifications. The researchers acknowledged their middle-class status, post-graduate qualifications while working in these communities marked by severe poverty. This self-awareness was essential to understand how our socio-economic status, race, gender and educational background could impact our interactions with participants and the data collected. For instance, being interviewed by a male, may have influenced the willingness of some of the female participants to disclose certain experiences. By actively reflecting on these factors during the weekly debriefing meetings and throughout the research process, the team aimed to mitigate potential biases and enhance confirmability of the study's findings. To ensure transferability, we provided detailed descriptions of the study context and participant characteristics, allowing readers to understand whether the findings might apply to their own settings.

19. The results section uses different formatting from the rest of the paper. Make that section "Results" and then go into the sample sociodemographic and characteristics

Thank you, reviewer 1 for picking this up. The formatting for the results section has been updated in line with the rest of the paper. The section heading has been updated from "findings" to "results"

20. What is the mean age?

The mean age was 36 years old. This detail has been added to the participant demographic information and it now reads:

The study included 20 participants, all experiencing different combinations of HIV, IPV and poor mental health. The participants were between the ages of 22-60, with a mean age of 36 years.

21.Do you have information on how long they've been living with HIV?

Yes, this information was provided by some of the participants, how long they had been living with HIV ranged from 2 years to 18 years. This detail has also been added to the paper under the socio-demographic information of the participants, and now reads:

Additionally of the participants who provided information on how long they had been living with HIV, they reported durations ranging from 2 to 18 years.

22. Give us a brief summary of the themes generated prior to discussing them. The four themes generated included x,y,z,a.

Thank you for this addition, Reviewer 1, at the beginning of the results section we have

**provided a summary of the themes. It now reads,**
**The study identified several factors among women experiencing the syndemic conditions that had an influence on their parenting practices. The four themes generated included mothers being separated from their children due to adverse circumstances, strained relationships with the fathers of their children, parental experiences of childhood trauma, including neglect, physical, and sexual abuse, and significant socio-economic challenges that hindered their capacity to meet the basic needs of their children.**

**23.The authors offer a little review of parenting but do not provide an understanding of parenting from the cultural perspective of South Africa. Consider revising this section of the background to add this. What is the cultural significance. It will help when the readers get to the results to contextualize the findings.**
**Thank you for this recommendation, Reviewer 1. We have updated the background section to include a detailed discussion on parenting from a South African cultural perspective to provide more context to the reader. This section reads:**
**The cultural significance of parenting in South Africa**
**Parenting practices in South Africa are deeply influenced by cultural norms and values.75 The extended family often plays a crucial role in parenting, with grandparents, aunts, uncles, and older siblings actively involved in the upbringing of children. 75–77 According to Statistics South Africa (2023), households with more than four children are more likely to have children cared for by extended family members (84.5%). Furthermore, the same survey found that a fifth of all children did not live with both their parents, and most children lived with only their mothers and other extended family members (42.0%).77 This collective approach to parenting is rooted in the African concept of "Ubuntu," which emphasizes compassion for others and the interconnectedness of all people.78 Traditional gender roles also influence parenting, with women generally seen as the primary caregivers responsible for day-to-day nurturing and education of children, while men provide financial support.76 Fragmented families in South Africa can be attributed to labour migration and low marriage rates especially among African women, who are also less likely to live with the child’s father if they are not married.76,79 The historical and socio-political context of South Africa, particularly the legacy of apartheid and ongoing socio-economic challenges, significantly shapes parenting practices.80 The psychological impact of the apartheid-era trauma has affected parenting as parents who experienced trauma may struggle with emotional regulation, influencing their ability to engage in consistent and nurturing practices.80 Additionally, during colonialism, corporal punishment was institutionalized as a method of maintaining order and discipline in both schools and homes.81 As a result, many South African homes today still view corporal punishment as integral to teaching children respect for authority and obedience.81,82 Despite the legislative ban on corporal punishment, many mothers continue to practice this form of parenting.83 A qualitative study found that parents disagreed with the law and held positive attitudes towards corporal punishment, viewing it as effective for disciplining children.82 Mothers often used corporal punishment to correct children's behaviour, lacking knowledge of alternative disciplinary methods.82 The use of corporal punishment is deeply embedded in cultural and historical contexts, passed down through generations as a normative practice.82**
**24.Which group is Halo part of? It’s unclear here. Page 15**
**Thank you for alerting us to this, Halo is part of the HIV-Mental health group, this has been made clearer in the paper**
**Halo, who was in her 40s from the HIV-Mental health group, openly shared how she disciplined her children,**
**25. Expand the lit review to discuss childhood trauma and the prevalence of sexual abuse**
**Thank you for this recommendation, Reviewer 1. We have expanded the literature review in the background section to include discussions on maternal childhood trauma and the prevalence of sexual abuse, this change is reflected in point 1, however for ease of reference it reads as**

**follows:**
**Other factors, such as adverse childhood experiences, can also increase the risk of the co-occurrence of the epidemics. Additionally, adverse maternal experiences in childhood not only elevate the risk of poor mental health in adulthood but also increase the likelihood of experiencing IPV and acquiring HIV 26,45, which, as previously noted, significantly impact parenting practices. Child sexual abuse is alarmingly prevalent in South Africa.46 A nationally representative cross-sectional study among 5631 households found that 9.99% of boys and 14.61% of girls reported experiencing sexual violence in their lifetime, with physical abuse, emotional abuse, and neglect strongly associated with sexual violence.46 Trauma from child abuse is a significant factor contributing to adult mental health challenges such as PTSD, depression, and anxiety.26,45,46The mental health challenges as a result of the abuse may increase the survivors' risk of being involved in violent relationships and as a result elevate their HIV risk. 26,47 Survivors of abuse often struggle with establishing healthy relationships due to the trauma, which can lead to low self-esteem, emotional dependency, and difficulty in setting boundaries. 26 This dynamic, combined with the impaired ability to negotiate safe sex, significantly heightens the risk of HIV infection among these individuals.47**
**26. I would encourage the authors to rethink the naming of the themes generated. Theme 1 living vs not living doesn't quite capture the essence of the process mothers are enduring for the betterment of their children. They're sacrificing the autonomy of raising their children due to financial strains. They are also embattled by gender inequalities- sexual violence.**
**Thank you, Reviewer 1. We understand your concern that the theme "Living vs Not Living with Children" may not fully capture the sacrifices and challenges mothers face for the betterment of their children amidst financial and social strains.**
**We appreciate your suggestion and have reconsidered the naming of this theme to better reflect the essence of the experiences described without losing the point being made regarding the women's living arrangements affecting their parenting practices. The revised theme name is Separated by adverse circumstances: The influence of living arrangements on parenting practices This theme more accurately conveys how the decision to live apart from their children, driven by various underlying factors, significantly influenced the mothers' parenting practices. The financial strains, sexual violence experiences are other underlying factors that influence parenting practices and they have been discussed later in the paper separately.**

**27. Provide an expanded discussion of cultural differences in child rearing and corporal punishment for Black mothers and caregivers. Provide a greater contextual foundation.**
**Thank you for this recommendation, Reviewer 1. We have incorporated an expanded discussion on cultural differences in child rearing and corporal punishment for Black mothers and caregivers into the background section (See point 1). This discussion provides context into the perceptions of African mothers on corporal punishment as well as the impact of the apartheid-era legacy on parenting practices. This section now reads:**
**The psychological impact of the apartheid-era trauma has affected parenting as parents who experienced trauma may struggle with emotional regulation, influencing their ability to engage in consistent and nurturing practices.80 Additionally, during colonialism, corporal punishment was institutionalized as a method of maintaining order and discipline in both schools and homes.81 As a result, many South African homes today still view corporal punishment as integral to teaching children respect for authority and obedience.81,82 Despite the legislative ban on corporal punishment, many mothers continue to practice this form of parenting.83 A qualitative study found that parents disagreed with the law and held positive attitudes towards corporal punishment, viewing it as effective for disciplining children.82 Mothers often used corporal punishment to correct children's behaviour, lacking knowledge of alternative disciplinary methods.82 The use of corporal punishment is deeply embedded in cultural and historical contexts, passed down through generations as a normative practice.82**

**28. Childhood trauma- this theme doesn't adequately capture the essence of the quotes. Again, gender inequality, parentification of girl children**
**We understand your concern that the theme "Childhood Trauma" may not fully capture the essence of the participants' experiences. After careful consideration, we agree that the theme could be more accurately named to reflect the breadth and impact of the participants' experiences. We have changed this theme to "Parental Experiences of childhood trauma and impact on parenting practices". We believe that renaming the theme to "Parental experiences of childhood trauma and the impact on parenting practices" better encapsulates the impacts these early adverse experiences have had on the participants and their parenting practices. This theme comprehensively covers the physical abuse, emotional and physical neglect, and childhood sexual violence that participants reported that they endured growing up. We also acknowledge that gender inequality and the parentification of girl children are significant aspects of the participants' experiences. These elements are indeed part of the broader context of childhood trauma and we feel can be addressed within the revised theme.**
**29. What is the grant that the women speak of? Provide the reader of an understanding of the welfare benefits.**
**Response**
**This is the social welfare grants provided by the government, for enhanced clarity we have added some context and explanation of the grants in the results section, it now reads:**
**Furthermore, participants reported that they relied on receiving social welfare grants from the government. These grants are means-tested and provide financial assistance to no- or low-income families and include the Child Support Grant (ZAR 510/ $27.77); the Older Person's Grant, which supports individuals over 60 years of age (ZAR 2090/ $113.79); the Disability Grant, which assists those aged 18-59 with disabilities (ZAR 2090/ $113.79); and the social relief of distress grant, which offers 3-month temporary aid for those in severe financial hardship (ZAR 350)/ $19.06).107 While these grants provided some financial relief, they were not always consistently paid.**
**30. Consider reviewing the quotes for readability; there are a few that should be edited for English grammar. Very minor but noticeable.**
**Thank you for bringing this to our attention. We have reviewed the quotes and made the necessary edits to improve their English and grammar for better readability.**
**31. Underlying theme is the gender-based violence impacting these women. Over protection is a manifestation of post-traumatic stress, hyper vigilance. This is vaguely discussed.**
**Response**
**We have now expanded the section to discuss in more detail how overprotection is a manifestation of post-traumatic stress and hypervigilance. The section now reads,**
**Research among women with experiences of childhood sexual abuse found that these mothers were more anxious, often experiencing post-traumatic stress that manifested as hypervigilance.128 The state of hypervigilance can lead to overprotective behaviours where mothers became excessively cautious and protective of their children.128 Parenting tasks and the development stages of the children can trigger memories and emotions related to their own abuse.129 For instance, when a child reaches the age at which the mother was abused, this can be triggering, leading to increased protectiveness, this fear can lead to behaviours such as constant monitoring, restricting the child's interactions with others and being overly cautious about physical touch and affection.129**
**32. The study discusses attachment theory, however, this theory wasn't discussed in the literature review- consider removing or address early on in the literature review. Same with family stress theory.**
**Thank you, Reviewer, 1, the reference to attachment theory as this was not initially discussed in the literature review has been removed this section now reads:**
**Additionally, our study revealed that mothers and caregivers generally did not report bonding**

challenges with their children, except in the IPV-Mental Health group, where participants reported these challenges. This finding is consistent with previous research which found that women that were traumatized by experiences of IPV reported more difficulties bonding with their children.119

The same has been done regarding the reference made to family stress theory, the section now reads:

In terms of parenting, our findings align with existing studies which have shown that economic hardship amplifies stress and inter-parental conflict 136. In our study this was particularly evident amongst the IPV-Mental Health group.

33. It may be a good idea to map out the conceptual framework to clearly delineate how variables have impacted each other. Poverty is a structural determinant whereas HIV, IV, and MH are psychosocial factors. Poverty can be exacerbated and underlying but not appear as a result of experiencing these things. upward mobility can be impacted. Yes, household income, yes. But not poverty

Thank you for this suggestion, Reviewer 1. We have taken this on board and incorporated a section that maps out the conceptual framework of the IPV, Mental Health, and HIV syndemic in the background, this has also been included in Point 1 above, however for ease of reference, please see below, this section now reads:

Conceptual Framework: IPV, Mental Health and HIV as a syndemic

Conceptual Framework: IPV, Mental Health and HIV as a syndemic

Existing literature confirms the frequent co-occurrence of the IPV, poor mental health and HIV epidemics.6–9 The relationship between HIV and poor mental health is bidirectional, meaning that HIV can lead to poor mental health outcomes, and poor mental health can increase the risk of acquiring HIV. 10,11 The high prevalence of poor mental health conditions among people living with HIV is often associated with poorer physical health, lower quality of health services, and poor adherence to antiretroviral treatment (ART). 12–14 Furthermore, having poor mental health can increase the risk of HIV acquisition through both direct and indirect mechanisms.15 Adolescents and adults with serious mental illnesses who are sexually active may be more likely to engage in high-risk behaviours, including inconsistent condom use, having multiple sexual partners, engaging in transactional sex and consuming alcohol before sex, which may increase the likelihood of unprotected sex.16–18 Additionally, IPV can precipitate mental health challenges such as PTSD, anxiety, depression, suicidal thoughts, and substance use disorder.19–23 Studies have shown that women experiencing IPV are at higher risk of developing these mental health challenges due to the chronic stress and trauma associated with ongoing abuse, as well as the possible isolation from social support systems.24–26 Conversely, poor mental health can make individuals more susceptible to IPV due to factors such as low self-esteem, emotional dependency, and difficulty recognizing abusive behaviours. 27,28Moreover, the relationship between IPV and HIV involves both biological and socio-cultural pathways.29 Biologically, forced or coercive sexual intercourse with an HIV-infected partner can lead to HIV and other sexually transmitted infections.30 Socio-culturally, violence and the fear of violence compromises the women's ability to negotiate condom use, which can lead to inconsistent condom use and thereby increase HIV risk. 31

An important aspect of syndemic theory is that structural and environmental factors impact the disease clustering and often exacerbate the syndemic conditions. 4,32 These include poverty, gender inequality, stigma, and lack of access to resources and services that could improve the health and wellbeing of individuals. 33,34 Financial stress and lack of resources can exacerbate mental health challenges, due to the higher levels of stress as a result of financial insecurity, job instability, and poor living conditions, which are significant risk factors for depression and anxiety.35 Furthermore, financial dependence on a partner can significantly reduce one's bargaining power within the household making it difficult to leave

an abusive situation.36 In addition, people living in poverty may engage in risky sexual behaviours to meet their basic needs, which can elevate their risk of contracting HIV.37,38 Cultural factors, such as gender norms and expectations, also influence the epidemics.39–41For instance in many societies, traditional gender roles can reinforce power imbalances, making women more vulnerable to IPV and less able to negotiate safe sex practices, increasing their risk of HIV.41 Additionally, stigma associated with IPV, poor mental health, and HIV can hinder individuals' willingness to seek help and adhere to treatment leading to further isolation, untreated conditions, and exacerbation of both physical and mental health challenges.42–44

Other factors, such as adverse childhood experiences, can also increase the risk of the co-occurrence of the epidemics. Additionally, adverse maternal experiences in childhood not only elevate the risk of poor mental health in adulthood but also increase the likelihood of experiencing IPV and acquiring HIV 26,45, which, as previously noted, significantly impact parenting practices. Child sexual abuse is alarmingly prevalent in South Africa.46 A nationally representative cross-sectional study among 5631 households found that 9.99% of boys and 14.61% of girls reported experiencing sexual violence in their lifetime, with physical abuse, emotional abuse, and neglect strongly associated with sexual violence.46 Trauma from child abuse is a significant factor contributing to adult mental health challenges such as PTSD, depression, and anxiety.26,45,46The mental health challenges as a result of the abuse may increase the survivors' risk of being involved in violent relationships and as a result elevate their HIV risk. 26,47 Survivors of abuse often struggle with establishing healthy relationships due to the trauma, which can lead to low self-esteem, emotional dependency, and difficulty in setting boundaries. 26 This dynamic, combined with the impaired ability to negotiate safe sex, significantly heightens the risk of HIV infection among these individuals.47

**34. Be careful of using causal language to describe qualitative data. Bottom 26. I don't think you can say poverty causes physical abuse but rather multistressed parents may invoke corporal punishment more than those without those stressors**

**Response:**

Thank you, Reviewer 1. We agree with your concern regarding the use of causal language in describing qualitative data. In our manuscript, we had indicated this by stating, "economic strain fosters increased parental stress, heightening the risk of poor mental health and, consequently, the adoption of harsher parenting practices (Ho et al., 2022; Wahlbeck et al., 2017)." In response to your suggestion, we have made it clearer to readers that poverty is a risk factor. The section has been edited to read:

This economic strain fosters increased parental stress, heightening the risk of poor mental health and, consequently, the adoption of harsher parenting practices.136,138 A review on the relationship between poverty and child abuse and neglect found that poverty is a significant risk factor for child maltreatment.139 Both familial and community-level poverty, as well as economic insecurity, have been strongly associated with an increased risk of child abuse and neglect.139 Economic hardships can lead to psychological distress, relationship problems, and disrupted parenting, which in turn can increase the likelihood of child abuse.139

**35. The question about disciplining the children wasn't asked in the context of stress and resolving conflict in the home thus the inference made is out of the context of the research question and aims.**

Thank you, Reviewer 1, for highlighting this issue. The question about disciplining the children aligns with the research question and aims by addressing how the relationship with the partner impacted the women's parenting. The stress and conflict resulting from being in an abusive relationship did emerge in our study and influenced how parents related to their children. This was not clearly reflected in the results section initially, but we have since corrected this oversight and added relevant quotes. The revised section now reads:

When it came to parenting in both the HIV-Mental Health and IPV-Mental Health groups, we found that participants often used corporal punishment as a means of disciplining their children. Participants in the IPV-Mental health group also shared that their experiences of IPV affected how they disciplined their children. Despite both groups using corporal punishment as a means of discipline there was a difference in how they described their relationship with the children. The mothers and caregivers from the IPV-Mental Health group reported an inability to connect with their children and a sense of detachment and dissatisfaction with parenting, Phoenix shared her dissatisfaction with being a parent, stating,
"I wouldn't want to be a parent if I had a choice. Nothing makes me happy about being a parent [laughs]. To be honest, I just don't find joy in being a parent."
Phoenix further described how her abusive relationship affected her interactions with her son, sharing,
"When I was in that abusive relationship, my son would try and speak to me, I would just insult him. I would get pissed off by my partner and take it out on the closest person to me and usually, it was my son he would do a small thing, like spill water, and I would overreact. I realised that the way I was hitting him was not normal. I would take out a lot of pain on
36. Address the translation in the methods section
Response
Thank you, reviewer 1, the translation process and challenges have now been moved to the methods section.

37. The study is also about supportive family members but does not delve into social support. Revise that as it was not the main focus of this study.
Response:
The discussion on social support has been removed, and the section under implications now discusses the importance of support from family members specifically. This change aligns the focus of the study with its findings and emphasizes the significance of family support in relation to parenting practices.

Reviewer 2
Limitations point 2 could be more specific as "arbitrary decisions" is unclear. Perhaps something like "Although validated scales for MH were used to identify participants with MH this may have introduced some arbitrary decisions being made."
Thank you, reviewer, 2, the second point on limitations has been edited for better clarity, it now reads:
Although validated scales for mental health were used to identify participants with poor mental health, this approach may have included individuals who endorsed symptoms but did not meet the strict cut-off criteria for a specific mental health challenge. This could have led to some misclassification.
In parts, this is referred to as a narrative study. In others, as an arts-based one. Please make consistent.
The study is a narrative study that used arts-based methods as a data collection technique. Narrative research focuses on understanding and interpreting the stories and experiences of participants. In this study, we used arts-based methods to collect data, utilizing creative techniques to elicit deeper insights and reflections. By combining these approaches, we aimed to capture a rich, nuanced understanding of the participants' experiences.
We have made this distinction clearer in the abstract and it now reads,
A qualitative research design using a narrative approach with in-depth interviews and supported by arts-based methods.

We have also clarified this in the data collection section to reads,
The in-depth interviews were supported through the use of arts-based methods

**Thematic analysis, particularly as described in the ms, seems insufficient for identifying underlying mechanisms for syndemics from arts-based, narrative interviews. The risk of overinterpretation seems very present. Further, to this point, I don't see mechanisms identified in the Results. I see stories, which fits the methodological approach of narrative arts-based work. I recommend removing reference to mechanisms, as the methods and results don't support this claim**
**Response:**
**Thank you, Reviewer 2, we agree with the suggestion, to this end we have removed reference to mechanisms and have rather spoken about the factors that may influence parenting practices among women living with the syndemics.**
**There are numerous acronyms that could confuse a reader. I recommend spelling out "mental health" and "South Africa" each time and encourage the authors to further trim the amount of acronyms.**
**Response:**
**Thank you, Reviewer 2, for this feedback. We acknowledge that the numerous acronyms can be confusing for readers. In response to your suggestion, we have spelled out "mental health" and "South Africa" throughout the paper to enhance clarity and other acronyms have also been reduced such as CSA has been spelt out each time to child sexual abuse, MSM to men who have sex with men.**
**The manuscript would benefit from careful proofreading as there are repeated words (e.g., "syndemic syndemics" in the Abstract), "data" treated as a singular term, and other issues (e.g., repeating "in-depth" twice in a single sentence).**
**- The in-text citations and references don't appear to be in Journal style.**
**- Some claims are missing citations (e.g., page 7, lines 12-14).**
**Response:**
**Thank you, Reviewer 2, for alerting us to these issues. While we did proofread the manuscript, we may have missed some errors. We have now carefully proofread and made the necessary edits to improve comprehension. The repeated words, incorrect treatment of "data" as a singular term, and other similar issues have been corrected. Additionally, we have ensured that all in-text citations and references adhere to the journal's style and added citations where claims were previously unsupported.**

## VERSION 2 – REVIEW

| | |
|---|---|
| **REVIEWER NAME** | ***Mangum, Laurenia*** |
| **REVIEWER AFFILIATION** | University of Illinois Chicago |
| **REVIEWER CONFLICT OF INTEREST** | Na |
| **DATE REVIEW RETURNED** | 23-Aug-2024 |

| | |
|---|---|
| **GENERAL COMMENTS** | Well done thoughtfully responding to the reviewer's comments. |

| | |
|---|---|
| **REVIEWER NAME** | ***Eaton, Andrew*** |
| **REVIEWER AFFILIATION** | University of Regina, Faculty of Social Work - Saskatoon Campus |

| | |
|---|---|
| **REVIEWER CONFLICT OF INTEREST** | Na |
| **DATE REVIEW RETURNED** | 21-Aug-2024 |

| | |
|---|---|
| **GENERAL COMMENTS** | The authors have addressed my prior concerns. |
